# Integrative single-cell analysis reveals *TRIM31^+^* colorectal tumor cells orchestrating macrophage crosstalk within the cancer-immunity regulome

**DOI:** 10.3389/fimmu.2026.1848654

**Published:** 2026-05-29

**Authors:** Yunxuan Shi, Xiaodong Yang, Mowen Xu, Qi Sun, Zihan Liu, Yuhan Yu, Hongling Jia, Wenyang Nie, Zhaidong Liu

**Affiliations:** 1 College of First Clinical Medicine, Shandong University of Traditional Chinese Medicine, Jinan, China; 2 Department of General Surgery, The First Affiliated Hospital of Anhui Medical University, Hefei, Anhui, China; 3 Henan Medical University, Xinxiang, Henan, China; 4 School of Clinical Medicine, Jiujiang University, Jiujiang, Jiangxi, China; 5 Second Clinical Medical College, Nanchang University, Nanchang, Jiangxi, China; 6 Department of Oncology, Affiliated Hospital of Shandong University of Traditional Chinese Medicine, Jinan, China

**Keywords:** Cancer-immunity regulome, colorectal cancer, single-cell RNA sequencing, TRIM31, tumor microenvironment

## Abstract

**Background:**

Colorectal cancer (CRC) continues to exhibit a high rate of both incidence and mortality, and most patients respond limitedly to immunotherapy. Increasing evidence suggests that tumor progression is governed by complex interactions between tumor cells (TCs) and the immune microenvironment. However, the multi-layered regulatory mechanisms underlying these interactions remain poorly understood.

**Methods:**

Single-cell RNA sequencing profiles sourced from GEO were merged for subsequent integrative analysis. Leveraging Seurat, we carried out data quality filtering, dimensionality reduction, clustering, and cell annotation, while Harmony was applied to correct batch effects. Malignant cells were identified using inferCNV. Functional characteristics were analyzed using various enrichment analyses, and pseudotime analysis was conducted using CytoTRACE, Monocle, and Slingshot. Communication between cells and transcriptional regulatory circuitry were constructed using CellChat and SCENIC. The critical factor *TRIM31* was confirmed through *in vitro* experiments. Furthermore, prognostic models were constructed based on TCGA data, and analyses of immune infiltration and drug sensitivity were performed.

**Results:**

The TCs were further classified into six subtypes, among which C4 *TRIM31^+^* TCs were enriched in tumor tissues, exhibited an immune-related phenotype, and showed significant enrichment in hallmark pathways. Integrative analysis suggested potential crosstalk between these cells and macrophages via MIF and GALECTIN signaling pathways. Silencing *TRIM31* significantly reduced the proliferative and invasive capacities of CRC cells. Moreover, the *TRIM31^+^* TCs risk group model constructed based on C4 *TRIM31^+^* TCs demonstrated effective prognostic stratification, especially for the high-risk group displayed enhanced immunosuppression and differential drug sensitivity.

**Conclusion:**

Overall, we characterized a comprehensive cancer-immune regulatory landscape involving transcriptional programs, intercellular crosstalk, and tumor evolution dynamics. Within this context, a distinct subtype of C4 *TRIM31^+^* TCs exhibiting immunoregulatory features was characterized, suggesting their involvement in shaping the cancer immunity landscape of CRC. Collectively, these results established a foundation for the identification of immune-associated biomarkers and offered insights into the prediction of immunotherapeutic efficacy and resistance.

## Introduction

Colorectal cancer (CRC) is a malignant tumor of the digestive tract arising from the colorectal mucosal epithelium, with both its incidence and mortality ranking among the highest worldwide (1). Global cancer statistics show that in 2020, there were approximately 1.93 million new cases and 930,000 deaths from CRC worldwide, representing 10.0% of global cancer incidence and 9.4% of cancer-related mortality, respectively (2). In China, with the westernization of lifestyles and the rapid aging of the population, the incidence and mortality rates of CRC have continued to rise, posing a substantial public health challenge and threatening overall population health (3, 4). In the past few years, significant clinical efficacy of immune checkpoint inhibitors has been observed in microsatellite instability-high/mismatch repair deficiency CRC, but the majority of microsatellite stable/mismatch repair-proficient patients show very limited responses to existing immunotherapeutic approaches (5, 6). Growing evidence suggests that immunotherapeutic resistance is shaped not by isolated molecular events, but by complex regulatory interactions between tumor cells (TCs) and the immune milieu. Together, these interactions define the cancer-immunity regulome, a coordinated network underlying immune dysfunction and tumor escape mechanisms.

Colorectal mucosal epithelial cells, as the origin cells of tumors, undergo malignant transformation under the gradual accumulation of genetic and epigenetic variations. Through gaining traits like excessive proliferation, resistance to cell death, and the ability to invade and metastasize, they eventually become TCs (1, 7). These transformed malignant cells not only serve as the core driving force for disease progression, but also exhibit specific biological behaviors that are regulated by both their intrinsic molecular characteristics and the tumor microenvironment (TME). Within the TME, stromal cells, endothelial cells (ECs), and immune cells-such as T cells, B cells, macrophages, and dendritic cells-interact to shape tumor behavior. And regulates the fate of TCs through various mechanisms such as direct killing, antigen presentation, and cytokine secretion (8). Among immune cells in the TME, tumor-associated macrophages (TAMs) are particularly abundant. They originate from peripheral blood mononuclear cells and exhibit high phenotypic plasticity. Microenvironmental signals can drive their polarization toward either M1 (classically activated) or M2 (alternatively activated) macrophages (9). Various stimulating signals in the TME induce the TAMs to undergo tumor-promoting functional transformation, thus promoting tumor proliferation, invasion, and metastatic progression (10). For instance, the conditioned medium of CRC cells can induce macrophages to exhibit a mixed M1/M2 phenotype. Moreover, by regulating the epithelial-mesenchymal transition process, it can enhance the migration and invasion of tumor cells (11). Therefore, a comprehensive investigation into the molecular mechanisms through which TCs regulate the immune microenvironment can provide important theoretical basis for discovering new immunotherapy targets.

*TRIM31* plays important roles in inflammatory responses, innate immunity, and tumor development. As a post-translational modification regulatory factor, *TRIM31* affects the stability, activity or interaction of substrate proteins by mediating their ubiquitination, thereby participating in various biological processes (12, 13). Increasing data indicate a strong link between abnormal *TRIM31* levels and tumor development across various cancers. Studies have shown that *TRIM31* enhances the stability of YBX1 by triggering its K63-linked ubiquitination, thereby promoting the mRNA stability of oncogenic target genes. It serves as a key mediator in the transition from inflammation to cancer and in driving CRC progression (14). Furthermore, *TRIM31* participates in the positive feedback regulation of the NF-κB signaling pathway. By promoting the nuclear entry of P65, it further activates its own transcription, thereby influencing the inflammatory microenvironment and metastatic potential of TCs (14, 15). Recent research has revealed that elevated *TRIM31* expression across multiple cancers is strongly associated with immune cell infiltration and resistance to immunotherapy (16). Given its multiple functions in tumor biology, elucidation of *TRIM31* and its complex regulatory mechanisms will aid in elucidating CRC pathogenesis while providing a conceptual framework for next-generation treatment strategies.

The development of single-cell RNA sequencing (scRNA-seq) technology enables the analysis of interaction networks between TCs and immune cells at single-cell resolution. Therefore, in this study, the first three tumor and adjacent non-tumor tissue pairs from CRC patients at the GEO database (GSE231559) were selected to systematically analyze the tumor’s immune milieu. Based on the data analysis, the distribution patterns and proportional composition of principal cell types within the TME were systematically characterized, and TCs with malignant potential were accurately identified using copy number variation (CNV) scores. TCs were further subjected to secondary clustering to comprehensively examine their composition, as well as the metabolic and functional pathway preferences of different subtypes, and the cellular state trajectories were inferred through pseudotime analysis. On this basis, a potentially key subtype, C4 *TRIM31^+^* TCs, was identified through intercellular communication analysis, which may contribute to CRC immune escape. The subtype’s transcriptional regulatory network was analyzed in greater detail, highlighting and visualizing the top 5 transcription factors (TFs). Additionally, the top 100 TFs were analyzed at the bulk transcriptome level to construct a prognostic model, associations with clinical outcomes, patterns of immune infiltration, and sensitivity to therapeutics were evaluated. This study sought to uncover the interaction mechanisms between CRC TCs and the immune system at integrative single-cell analysis, offering a novel perspective on CRC’s immune regulatory network.

## Materials and methods

### scRNA-seq data collection and preparation

scRNA-seq data were obtained from the Gene Expression Omnibus (GEO; accession GSE231559, https://www.ncbi.nlm.nih.gov/geo/). A subtype of samples, including CRC tissues and adjacent normal tissues, was selected and integrated for subsequent analyses. Following the import of raw gene-expression data into R (version 4.4.1), downstream analyses were carried out using the Seurat package (version 4.3.0). After initial quality control filtering, the DoubletFinder package (version 2.0.3) was applied to identify and remove potential doublets. Gene expression matrices were then processed using the “NormalizeData” function for logarithmic normalization, followed by identification of the 2,000 most highly variable genes using the “FindVariableFeatures” function. The data were subsequently scaled using the “ScaleData” function. Principal component analysis (PCA) was performed, and batch effects were mitigated using the Harmony package (version 0.1.1). The top 30 principal components were retained for downstream analyses, including clustering and UMAP-based visualization. These parameter settings were determined with reference to previously published studies (17–19).

### Measurement of cellular CNV levels

CNV profiles were inferred using the infercnv package (version 1.17.0). Fibroblasts were designated as the reference population to evaluate chromosomal copy number alterations in CRC cells, enabling the distinction between non-malignant cells and malignant TCs (20, 21).

### Cell type identification

The first 30 principal components were retained for downstream dimensionality reduction and clustering analyses. Cell distributions were subsequently visualized in a two-dimensional space using the Uniform Manifold Approximation and Projection (UMAP) algorithm to facilitate cell type annotation, followed by secondary clustering for subtype identification. Marker genes were determined based on published studies and the CellMarker database (http://xteam.xbio.top/CellMarker/) to support accurate annotation (22, 23).

### Analysis of differentially expressed genes (DEGs) and their functional enrichment

DEGs for each cell type were identified using the Wilcoxon rank-sum test implemented in the “FindAllMarkers” function (24, 25). Functional enrichment analyses, including Gene Ontology (GO), were subsequently performed using the clusterProfiler (version 4.6.2) and SCP (version 0.4.8) packages. All analysis parameters and thresholds were determined according to previously published studies (26–32).

### AUCell score analysis

In this study, the activity of CRC-associated gene sets was assessed using SCGMT (version 0.0.3) implemented within the AUCell framework (https://github.com/ZhaoLabs-SJTU/scgmt) (33–35).

### Pseudotime analysis

Cellular stemness was quantified and differentiation dynamics were inferred using the CytoTRACE package (version 0.3.3). Pseudotime trajectories were subsequently visualized with the Monocle package (version 2.24.0), utilizing the “plot_cell_trajectory” function to order cell subtypes along developmental progression. In addition, lineage relationships and corresponding pseudotemporal paths were reconstructed. The “getCurves” function in the Slingshot package (version 2.6.0) was utilized for trajectory inference (36, 37).

### Single-cell regulatory network inference and clustering analysis

The Python tool SCENIC (version 1.3.1) constructs single-cell regulatory networks to explore the TFs with the most significant expression differences in each subtype. The three steps of this process are detailed in the references (38).

### Analysis of intercellular communication

Intercellular communication networks in CRC were inferred and visualized with the CellChat R package (version 1.6.1), incorporating functions such as “netVisualDiffInteraction”, “IdentifyCommunicationPatterns”, and “netVisual_circle”. This approach enabled the analysis of ligand-receptor interaction patterns and facilitated the investigation of coordinated regulatory interactions among different cell types (39–42).

### Hallmark score

The activity of TC subtypes across 50 hallmark cancer pathways from the MSigDB database (version 7.5.1) was evaluated using the ssGSEA algorithm implemented in the GSVA package (43, 44).

### Culture conditions for the cell lines used

SW480 and HCT116 human CRC cell lines were acquired from the ATCC (Manassas, VA, USA). Cell line authentication was confirmed by short tandem repeat profiling, and routine mycoplasma testing was performed prior to experimentation. SW480 cells were maintained in Leibovitz’s L-15 medium (Gibco, Thermo Fisher Scientific; Cat# 11415-064) supplemented with 1% penicillin-streptomycin (Gibco, Cat# 15140-122) and 10% fetal bovine serum (FBS; Gibco, Cat# 10099-141), and cultured at 37 °C in a CO_2_-free environment according to ATCC recommendations. In contrast, HCT116 cells were cultured in McCoy’s 5A medium (Gibco, Cat# 16600-082) containing 10% FBS and 1% penicillin-streptomycin, and maintained at 37 °C in a humidified incubator with 5% CO_2_. Cells were passaged every 2–3 days, and only cells between passages 3 and 10 were used for subsequent experiments (45–49).

### siRNA transfection

Two distinct small interfering RNAs targeting human *TRIM31* (si-*TRIM31*#1 and si-*TRIM31*#2) and a non-targeting negative control (si-Ctrl) were synthesized by GenePharma (Shanghai, China). The corresponding sequences were provided in **Supplementary Table 1**. Cells were seeded into 6-well plates and transfected at approximately 50-70% confluence using Lipofectamine RNAiMAX Transfection Reagent (Invitrogen, Thermo Fisher Scientific, Cat# 13778075) according to the manufacturer’s instructions.. The final siRNA concentration was 50 nM. Following a 6-hour incubation, the culture medium was replaced with fresh complete medium, and cells were harvested 48 hours post-transfection for subsequent experiments. The experimental groups included:

- si-Ctrl.
- si-*TRIM31*#1.
- si-*TRIM31*#2.

The use of two independent siRNAs reduced off-target effects and ensured specific attribution of the observed phenotype (50–54).

### RNA extraction and quantitative real-time PCR

Total RNA was isolated using TRIzol reagent (Invitrogen, Thermo Fisher Scientific; Cat# 15596026) in accordance with the manufacturer’s protocol. RNA concentration and purity were assessed using a NanoDrop 2000 spectrophotometer. Subsequently, cDNA was synthesized with the PrimeScript RT Reagent Kit (Takara, Cat# RR037A). qRT-PCR was carried out on a QuantStudio 6 Flex system (Applied Biosystems) using SYBR Green PCR Master Mix (Cat# A25742). The relative expression level of *TRIM31* was calculated using the 2^-^ΔΔCt method with GAPDH as the internal control. Primer sequences are provided in **Supplementary Table 1**. All experiments were conducted with three independent biological replicates, each performed in triplicate (55–57).

### Proliferation analysis of cells

Cell proliferation was evaluated using the Cell Counting Kit-8 (CCK-8; Dojindo Laboratories, Cat# CK04) according to the manufacturer’s instructions. Transfected cells were seeded into 96-well plates at a density of 2-3 × 10³ cells per well, with five replicate wells assigned to each group. At 0, 1, 2, 3, and 4 days post-seeding, 10 μL of CCK-8 reagent was added to each well, followed by incubation at 37 °C for 1–2 hours. Absorbance at 450 nm (OD450) was measured using a BioTek Synergy H1 microplate reader, and cell proliferation curves were subsequently generated from these values (58–60).

### Evaluation of colony-forming ability

For colony formation assays, transfected cells were seeded into 6-well plates at a density of 500–800 cells per well and cultured for 10–14 days, with the medium replaced every 3 days. Colonies were subsequently fixed in 4% paraformaldehyde for 20 min and stained with 0.1% crystal violet for 15 min at room temperature. Only colonies containing more than 50 cells were counted under a light microscope. Representative images were recorded, and colony numbers were quantified based on three independent experiments (61).

### Transwell-based cell migration assay

Cell migration was evaluated using transwell chambers with 8-μm pore polycarbonate membranes (Corning, NY, USA; Cat# 3422). Forty-eight hours after transfection, cells were collected and gently resuspended in serum-free medium for subsequent experiments. A total of 5 × 10^4^ cells were suspended in 200 μL of serum-free medium and seeded into the upper chamber, while 600 μL of complete medium containing 10% FBS was added to the lower chamber to serve as a chemoattractant.

After 24 h of incubation at 37 °C, cells that had not migrated and remained on the upper surface were gently wiped away using cotton swabs. Cells that had migrated to the lower surface were fixed in 4% paraformaldehyde and subsequently stained with crystal violet. The number of migrated cells was then quantified by counting five randomly selected microscopic fields (62–65).

### Apoptosis analysis by flow cytometry

Apoptotic cells were assessed using the Annexin V-FITC/propidium iodide (PI) Apoptosis Detection Kit (BD Biosciences, San Jose, CA, USA; Cat# 556547) according to the manufacturer’s protocol. At 48 h post-transfection, cells were collected, washed twice with ice-cold phosphate-buffered saline, and resuspended in binding buffer. Subsequently, cells were incubated with Annexin V-FITC and PI for 15 min at room temperature in the dark. Apoptotic cells were analyzed using a BD FACSCanto II flow cytometer (BD Biosciences), and data were processed with FlowJo software (Tree Star Inc., Ashland, OR, USA). Early apoptosis was defined as Annexin V^+^/PI^-^, whereas late apoptosis was characterized by Annexin V^+^/PI^+^ staining. The overall apoptosis rate was calculated as the sum of early and late apoptotic cell populations (66, 67).

### CRC prognostic model construction

Patient-specific gene expression data were obtained from the TCGA CRC cohort. Candidate genes were initially screened using univariate Cox regression, followed by feature selection with the LASSO-Cox regression model implemented in the glmnet package (version 4.1.10). Multivariate Cox regression analysis was subsequently performed to determine regression coefficients, which were used to construct a prognostic risk score model for CRC. The risk score was calculated as follows: Risk score = ∑ (regression coefficient × gene expression level).

Survival differences between groups were evaluated using Kaplan-Meier analysis with the survival package (version 3.8.3) in R, and visualized using the “ggsurvplot” function. In addition, time-dependent receiver operating characteristic (ROC) curves were generated using the timeaROC package (version 0.4) to assess predictive accuracy. Based on independent prognostic factors, Cox proportional hazards nomograms were further constructed to estimate overall survival at 1, 3, and 5 years. The evaluation criteria for model performance were defined according to previously published studies. (68–72).

### Immunoassay of the CRC microenvironment

We used the ESTIMATE (version 1.0.13) and xCell (version 1.1.0) packages in R to calculate the matrix score, immune score, overall ESTIMATE score, and tumor purity. To further characterize the immunochromatographic test result of CRC samples, we combined the CIBERSORT algorithm (version 0.1.0) to quantitatively assess the relative abundance and infiltration level of 22 tumor-infiltrating immune cells. This method allowed for a detailed assessment of the enrichment of immune cells throughout the cohort (73–75).

### Genomic alteration and mutation profiling analysis

DNA sequencing data were analyzed to detect multiple types of genomic alterations, including single nucleotide variants (SNVs), insertions/deletions, CNVs and chromosomal translocations. SNV calling was performed using MuTect and VarDict, while CNV profiles were quantified with CNVkit based on predefined thresholds. For structural variation analysis, translocation events were identified using NovoBreak and Lumpy, requiring at least four supporting reads for reliable detection. Identified mutations were further categorized into six substitution types (C>A, C>G, C>T, T>A, T>C, and T>G). All genomic alterations were annotated and monitored using GENEKEEPER. Tumor mutational burden (TMB) was calculated based on SNVs with an allele frequency ≥10% (76).

### Drug-susceptibility analysis

Drug response predictions were performed using data from the Genomics of Drug Sensitivity in Cancer (GDSC) in combination with the pRRophetic R package (version 0.5) to estimate the half-maximal inhibitory concentration (IC50) for various compounds in tumor samples. To assess the reliability of these predictions, 10-fold cross-validation was conducted on the GDSC training set. Details regarding parameter settings and batch effect correction procedures were implemented according to previously published studies (77).

### Statistical evaluation

All experimental results are presented as mean ± standard deviation (SD). Statistical analyses were conducted using GraphPad Prism 9.0 (GraphPad Software, San Diego, CA, USA). Comparisons among multiple groups were performed with one-way ANOVA, followed by Tukey’s *post-hoc* test for pairwise comparisons (78). Statistical significance was defined as *P* < 0.05, high significance as *P* < 0.001, and very high significance as *P* < 0.0001 *(*79–83).

## Results

### The single-cell atlas of CRC revealed TCs identified based on CNVs and their TME heterogeneity

**Figure 1:**
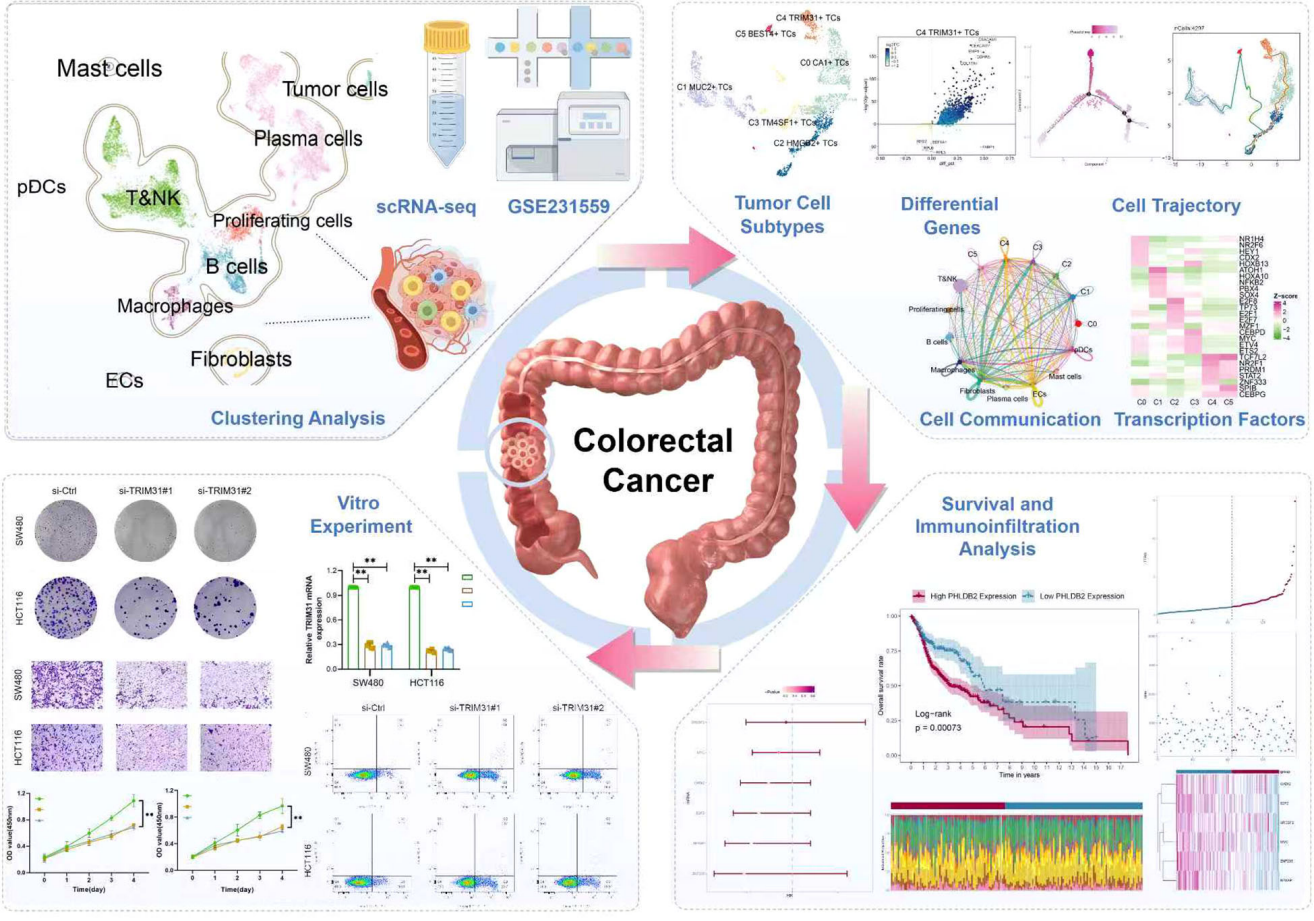
Workflow for single-cell transcriptomic analysis of the GSE231559 dataset and characterization of CRC tissue heterogeneity.

**Figure 2:**
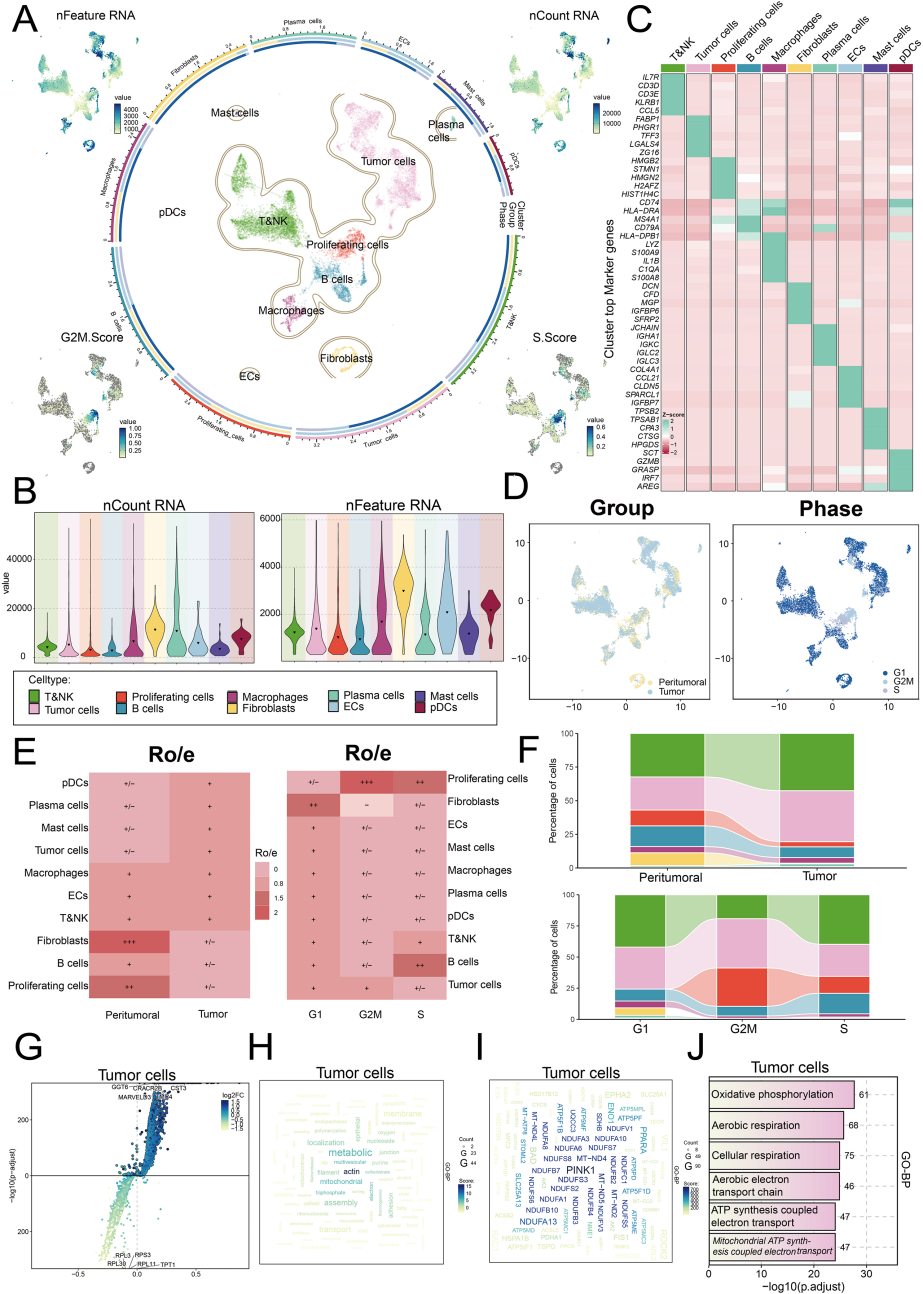
Cellular composition and functional characteristics of the CRC TME revealed by single-cell transcriptomics. **(A)** The UMAP plots showed the cluster distribution of all cells in the CRC TME. The plot showed cell type annotations, including pDCs, mast cells, T cells and NK cells, ECs, macrophages, fibroblasts, B cells, proliferating cells, TCs, and plasma cells. The four corner UMAP plots showed the expression distribution of quality control indicators: nFeature RNA (top left), nCount RNA (top right), G2/M.Score (bottom left), and S.Score (bottom right), with the color intensity indicating the corresponding score. **(B)** The violin plots showed the expression of quality control indicators for different cell types, including nCount RNA (left) and nFeature RNA (right). **(C)** The heatmap showed the top 5 DEGs for each cell type. **(D)** The UMAP plots showed the expression distribution of Group (peritumoral vs tumor) and phase (G1, G2/M, S). **(E)** The Ro/e preference analysis heatmaps showed the distribution preference of different cell types in the peritumoral and tumor groups (left), and the distribution preference in the G1, G2/M, and S phases (right). **(F)** The scale plots showed the changes in the proportion of cell types between groups (top) and the changes in their distribution within a phase (bottom). **(G)** The volcano plot showed DEGs in TCs, displaying key genes that were upregulated and downregulated, with colors labeled by log2FC values. **(H)** The word cloud plot illustrated the biological processes of functional enrichment in TCs. **(I)** The word cloud showed the genes that were functionally enriched in TCs. **(J)** The bar plot showed the results of GO-BP functional enrichment analysis of TCs, which significantly enriched oxidative phosphorylation, aerobic respiration, cellular respiration, etc.

Based on scRNA-seq of CRC data GSE231559, cellular composition and microenvironmental features were analyzed in depth for both CRC and adjacent tissues to reveal their underlying differences. The overall analysis workflow was illustrated in **Figure 1**. CRC was a malignant tumor derived from intestinal epithelial cells (7), in the figure, clusters 14 and 18 were identified as fibroblasts, whereas clusters 10, 11, 13, 15, 2, 22, 24, 5, and 9 were annotated as intestinal epithelial cells. To further delineate and investigate tumor heterogeneity, fibroblasts were selected as the reference baseline, as cancer-associated fibroblasts were recognized as stromal components of the tumor microenvironment and represent a heterogeneous, non-malignant cell population rather than cells of tumor origin (84). Using inferCNV analysis, we identified TCs, which corresponded to all intestinal epithelial cell clusters (**Supplementary Figures 1A, B**). The violin plot of CNV scores clearly demonstrated that intestinal epithelial cells (clusters 10, 11, 13, 15, 2, 22, 24, 5, and 9) exhibited significantly higher CNV levels compared to fibroblasts (clusters 14 and 18). These findings indicated a high degree of malignancy across all intestinal epithelial cell clusters, and therefore, these cells were collectively annotated as TCs (**Supplementary Figure 1C**). After performing quality control and correcting batch effects in the collected CRC tissues, we carried out dimensionality reduction and integrated high-quality filtered cells. Major cellular populations identified from the data consisted of plasmacytoid dendritic cells (pDCs), mast cells, T cells and natural killer (NK) cells, ECs, macrophages, fibroblasts, B cells, proliferating cells, TCs, and plasma cells. For quality control, the UMAP projections of nFeature RNA, nCount RNA, S.Score, and G2/M.Score were visualized in the four corners of the UMAP plot (**Figure 2A**), violin plots were used to show expression patterns of nFeature RNA and nCount RNA in various cells populations (**Figure 2B**), data quality and gene expression complexity were evaluated. Furthermore, we used heatmap to present the highest-expressed five DEGs in each cellular subtype population to verify the rationality and accuracy of different cell types (**Figure 2C**). Meanwhile, UMAP-based dimensionality reduction visualization was used to visualize the expression distribution characteristics across the different groups and corresponding cell cycle phases. Findings revealed that TCs were predominantly derived from the tumor group and were mainly in the G1 phase, whereas fibroblasts were primarily derived from the peritumoral group and also predominantly existed in the G1 phase (**Figure 2D**). In the Ro/e heatmaps, we can visually observe the bias of groups and phases in various cell types. In terms of group preference, pDCs, plasma cells, mast cells, and TCs were enriched in the tumor group, whereas fibroblasts, B cells, and proliferating cells were enriched in the peritumoral group. Regarding cell cycle phase, proliferating cells were more associated with the G2/M and S phases, while fibroblasts, in contrast, preferred the G1 phase (**Figure 2E**). In the proportion distribution plot of group, compared with the tumor group, T cells, NK cells, and TCs from peritumoral tissues all exhibited reduced proportions. In the map depicting phase distribution, the proportion of TCs and proliferating cells in G2/M phase was the largest compared with G1 and S phase, proliferating cells appeared to be less evident in the G1 phase. However, T cells and NK cells had the smallest proportion in G2/M stage (**Figure 2F**). It was well known that the tumor ecosystem consisted of malignant cells and non-malignant microenvironmental cells, within which tumor epithelial cells served as the evolutionary units that underwent transcriptional reprogramming under selective pressure and regulated the recruitment and function of immune cells through secreted signaling molecules (85, 86). Therefore, deciphering the intrinsic programs of TCs was critical for understanding their immune remodeling. We analyzed TCs using a volcano plot, which revealed that *GGT6*, *CRACR2B*, *CST3*, *MARVELD3*, and *TMC4* were the top 5 significantly upregulated genes (**Figure 2G**). Meanwhile, the word cloud illustrated that the biological processes enriched among the DEGs were primarily related to actin, metabolism, and mitochondria, and the enriched genes mainly included *PINK1*, *NDUFS3*, *MT-ND4*, and others (**Figures 2H, I**). According to the bar plot, TCs were predominantly concentrated in oxidative phosphorylation, the aerobic electron transport chain, aerobic respiration, ATP synthesis coupled electron transport, cellular respiration, and mitochondrial ATP synthesis coupled electron transport (**Figure 2J**). This indicated that TCs significantly depended on mitochondrial-driven oxidative phosphorylation for energy metabolism, suggesting that they met their high proliferation and biological function requirements by enhancing aerobic respiration and ATP synthesis.

### Single-cell subtyping of TCs and evaluation of their functional properties.

**Figure 3:**
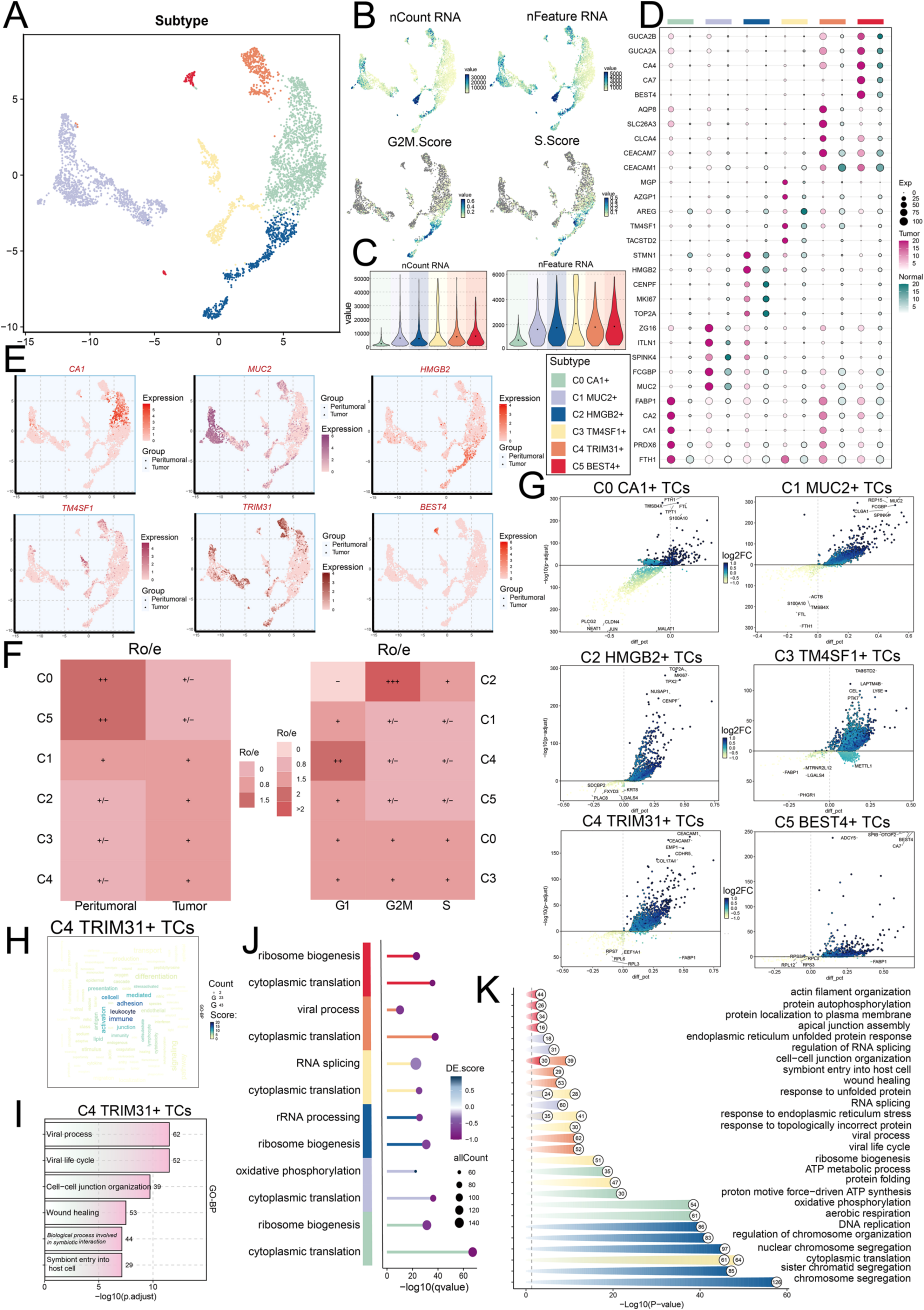
Identification, differential expression, and functional enrichment analysis of TC subtypes. **(A)** The UMAP plot showed the clustering of TC subtypes, and a total of 6 subtypes were identified: C0 *CA1^+^* TCs, C1 *MUC2^+^* TCs, C2 *HMGB2^+^* TCs, C3 *TM4SF1^+^* TCs, C4 *TRIM31^+^* TCs, and C5 *BEST4^+^* TCs. **(B)** The UMAP plots showed the expression distribution of quality control indicators nCount RNA, nFeature RNA, G2/M.Score, and S.Score for TC subtypes, with the color intensity indicating the corresponding score. **(C)** The violin plots showed the expression of nCount RNA and nFeature RNA in TC subtypes. **(D)** The bubble plot showed the top 5 DEGs of TC subtypes. **(E)** The UMAP plots showed the expression distribution of named genes in six TC subtypes, as well as the group distribution. **(F)** The Ro/e preference analysis heatmaps showed the distribution preference of different subtypes in the peritumoral and tumor groups (left), and the distribution preference in the G1, G2/M, and S phases (right). **(G)** The volcano plots showed DEGs in TC subtypes (C0-C5), highlighting key genes that were upregulated and downregulated, with color-coded log2FC values. **(H)** The word cloud illustrated the biological processes of functional enrichment of C4 *TRIM31^+^* TCs. **(I)** The bar plot showed the results of GO-BP functional enrichment analysis of C4 *TRIM31^+^* TCs, which were significantly enriched in viral process, viral life cycle, cell-cell junction organization, etc. **(J)** The lollipop plot showed the functional enrichment scores of each TC subtype. **(K)** The GO enrichment analysis showed the biologically enriched pathways of each TC subtype.

Therefore, we targeted TCs for further analysis and clustered them into six subtypes by dimensionality reduction, specifically, the identified TC subtypes include C0 *CA1^+^* TCs, C1 *MUC2^+^* TCs, C2 *HMGB2^+^* TCs, C3 *TM4SF1^+^* TCs, C4 *TRIM31^+^* TCs and C5 *BEST4^+^* TCs (**Figure 3A**). To achieve this, quality control was conducted, and UMAP plots were employed to visualize how nCount RNA, nFeature RNA, G2/M.Score, and S.Score were distributed across the dataset, while violin plots illustrated the expression patterns of nCount RNA and nFeature RNA (**Figures 3B, C**). At the same time, we used DEGs as the basis for classifying subtypes, and bubble plot was used to show the five most significantly DEGs in each subtype (**Figure 3D**). To better understand the expression patterns of the six genes associated with subtypes, we visualized their profiles. We employed UMAP for visualization (**Figure 3E**). Notably, in the Ro/e analysis, C0 *CA1^+^* TCs and C5 *BEST4^+^* TCs were preferentially enriched in the peritumoral group, whereas C2 *HMGB2^+^* TCs, C3 *TM4SF1^+^* TCs, and C4 *TRIM31^+^* TCs were preferentially enriched in the tumor group. C2 *HMGB2^+^* TCs were more associated with the G2/M phase, C1 *MUC2^+^* TCs, C4 *TRIM31^+^* TCs, and C5 *BEST4^+^* TCs, on the other hand, were mainly enriched in the G1 phase (**Figure 3F**). To gain deeper insights into the heterogeneity among TCs, we performed functional enrichment analysis of six subtypes. Using volcano plot analysis, the five most upregulated genes in C0 *CA1^+^* TCs were *TMSB4X*, *FTH1*, *TPT1*, *FTL*, and *S100A10* were identified. The five genes with the greatest upregulation in C1 *MUC2^+^* TCs were *REP15*, *MUC2*, *CLCA1*, *FCGBP* and *SPINK4*. *TOP2A*, *MKI67*, *TPX2*, *NUSAP1* and *CENPF* were the top 5 up-regulated genes in C2 *HMGB2^+^* TCs. *TACSTD2*, *LAPTM4B*, *CEL*, *LY6E* and *PTK7* were the top 5 up-regulated genes in C3 *TM4SF1^+^* TCs. The top 5 up-regulated genes of C4 *TRIM31^+^* TCs were *CEACAM1*, *CEACAM7*, *EMP1*, *CDHR5* and *COL17A1*. The top 5 genes significantly up-regulated by C5 *BEST4^+^* TCs were *ADCY5*, *SPIB*, *OTOP2*, *CA7* and *BEST4* (**Figure 3G**). Notably, among the DEGs enriched biological processes displayed in the word cloud map, C4 *TRIM31^+^* TCs showed significant involvement in viral processes, viral life cycle, cell-cell junction organization, wound healing, symbiotic interactions, and symbiont entry into host cells (**Figure 3H**). Moreover, C4 *TRIM31^+^* TCs were significantly displaying significant involvement in biological processes connected to viral processes, viral life cycle, cell-cell junction organization, wound healing, biological processes involved in symbiotic interactions, and symbiont entry into host cells (**Figure 3I**). The lollipop plot showed obvious functional heterogeneity among the different TC subtypes (**Figure 3J**). Functional enrichment via GO analysis indicated that TCs showed significant involvement in chromosome segregation, sister chromatid segregation, and cytoplasmic translation (**Figure 3K**).

### Analysis of tumor-related signaling pathway characteristics and quasi-temporal development trajectory of C4 *TRIM31^+^* TCs

**Figure 4:**
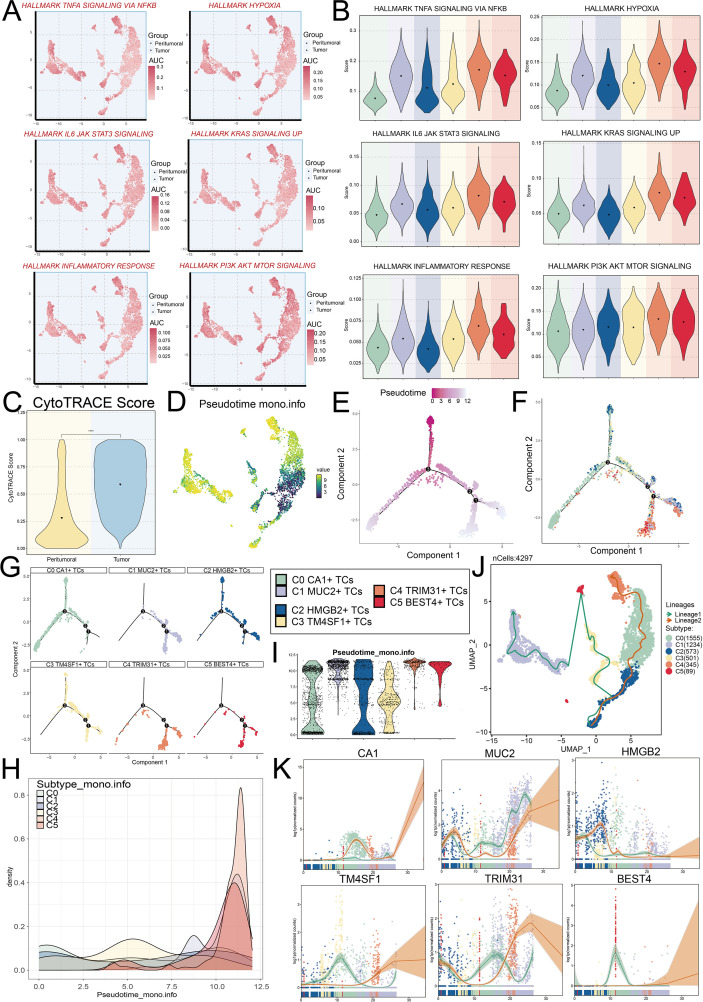
Pathway activity, stemness characteristics, and pseudotime differentiation trajectory of TCs. **(A)** The UMAP plots showed the enrichment distribution of key hallmark pathways (TNFA signaling via NFκB, IL6 JAK STAT3 signaling, hypoxia, KRAS signaling up, inflammatory response, and PI3K AKT MTOR signaling pathways). **(B)** The violin plots showed the enrichment of key hallmark pathways (TNFA signaling via NFκB, IL6 JAK STAT3 signaling, hypoxia, KRAS signaling up, inflammatory response, and PI3K AKT MTOR signaling pathways) in TC subtypes. **(C)** The violin plot showed the expression of CytoTRACE scores in TCs in the peritumoral and tumor groups. **(D)** The UMAP plot showed the pseudotime of TCs inferred by Monocle. **(E)** The pseudotime trajectory plot showed the differentiation trajectory of TCs, with black lines indicating the differentiation paths and numbers marking the differentiation nodes. **(F, G)** The pseudotime trajectory plots showed the expression distribution of each TC subtype along the differentiation trajectory. **(H)** The ridge plot showed the expression of each TC subtype along the pseudotime axis. **(I)** The violin plot showed the pseudotime distribution of each TC subtype. **(J)** Two differentiation trajectories, lineage1 and lineage2, were calculated using Slingshot. **(K)** The dynamic trend plots showed the expression changes of named genes (*CA1*, *MUC2*, *HMGB2*, *TM4SF1*, *TRIM31*, and *BEST4*) along the pseudotime axis, with different colored lines representing different lineages.

To further dissect the correlation between C4 *TRIM31^+^* TCs and CRC, hallmark score analysis was performed on the top 50 tumor-associated pathways. Among them, C4 *TRIM31^+^* TCs were significantly enriched in hypoxia, IL6 JAK STAT3 signaling, KRAS signaling up, inflammatory response, TNFA signaling via NFκB, and PI3K AKT MTOR signaling pathways. UMAP plots were employed to visualize the expression distribution and violin plots to show the expression, respectively (**Figures 4A, B**). We then performed a pseudo-temporal analysis of the six subtypes. In CytoTRACE, we visualized the expression of group with violin plot and found that the tumor group was significantly higher than peritumoral group, which was statistically significant (**Figure 4C**). We then applied Monocle algorithm to reveal continuous developmental gradients in TC subtypes by UMAP-based quasi-temporal visualization (**Figure 4D**). At the same time, the trajectory we established showed a branching structure, containing four different developmental paths, which generally progressed from the top to the bottom and from the left to the right corners (**Figure 4E**). Subtype-level analysis revealed significant temporal separation along the pseudotime. Specifically, enrichment of C0 *CA1^+^* TCs, C2 *HMGB2^+^* TCs, and C3 *TM4SF1^+^* TCs was observed in the early stage of the pseudo-timing, by comparison, these subtypes-C1 *MUC2^+^*, C4 *TRIM31^+^*, and C5 *BEST4^+^* TCs-were concentrated primarily in the late pseudotime phase (**Figures 4F, G**). The ridge plot supported the observation that C0 *CA1^+^* TCs and C2 *HMGB2^+^* TCs mainly occupied the initial portion of the pseudotemporal sequence, while C1 *MUC2^+^* TCs, C4 *TRIM31^+^* TCs, and C5 *BEST4^+^* TCs were biased toward the terminal phase (**Figure 4H**). Consistent with the violin plot data, C4 *TRIM31^+^* TCs were preferentially distributed in the late pseudo-timeline in the inferred developmental trajectory (**Figure 4I**). To further resolve the lineage relationships, we employed Slingshot and identified two distinct lineages. Lineage 1 originated from C2 *HMGB2^+^* TCs and differentiated successively into C1 *MUC2^+^* TCs. Lineage 2 shared the same origin as lineage 1 but differentiated successively into C4 *TRIM31^+^* TCs (**Figure 4J**). Specifically, our dynamic gene expression analysis along the pseudo-time series revealed distinct temporal expression patterns, with *TRIM31* showing higher expression in the late pseudotime stage (**Figure 4K**).

### C4 *TRIM31^+^* TCs and macrophages mediate bidirectional communication through MIF and GALECTIN signal axis

**Figure 5:**
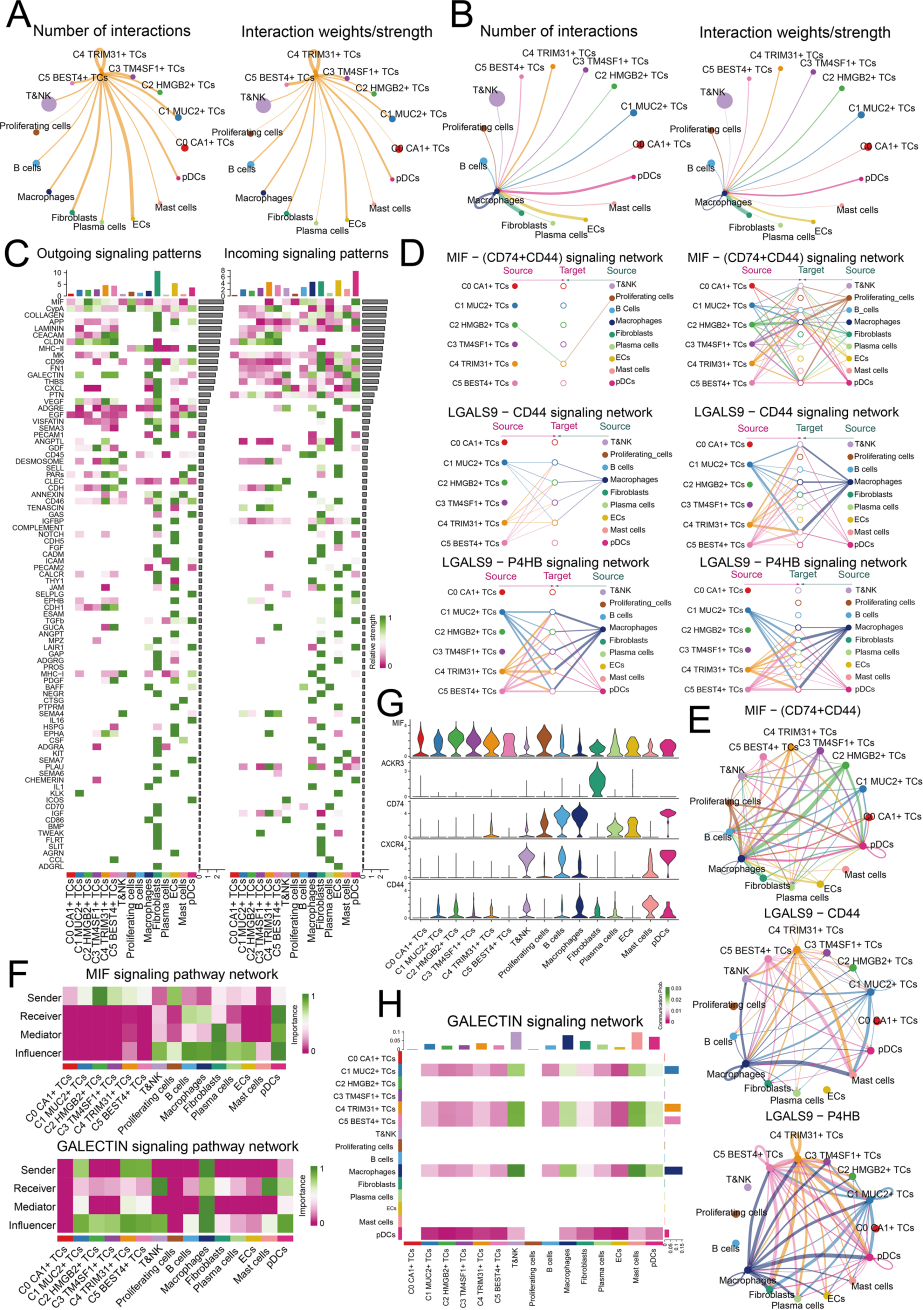
Cell–cell communication networks driven by C4 *TRIM31^+^* TCs and MIF and GALECTIN signaling pathways. **(A)** The circular plots of cell-cell interactions with C4 *TRIM31^+^* TCs as the signaling source were shown. The left panel showed the number of interactions between different cell types, and the right panel showed the interaction weight/strength. The node size represented the number of cells, and the line width corresponded to the interaction strength; the colors distinguished the signaling source cell types. **(B)** The circular plots of cell-cell interactions with macrophages as the signaling source were shown. The left panel showed the number of interactions between different cell types, and the right panel showed the interaction weight/strength. The node size represented the number of cells, and the line width corresponded to the interaction strength; the colors distinguished the signaling source cell types. **(C)** The heatmaps showed the outgoing signaling patterns (left) and incoming signaling patterns (right), displaying the relative signaling strength of different cell types across various signaling pathways. **(D)** The hierarchical plots showed the cell-cell communication of the MIF-(CD74+CD44), LGALS9-CD44, and LGALS9-P4HB signaling pathways. **(E)** The circular plots showed the cell-cell communication networks of the MIF-(CD74+CD44), LGALS9-CD44, and LGALS9-P4HB signaling pathways. **(F)** The centrality score plots showed the communication centrality scores of each cell type as sender, receiver, mediator, and influencer in the MIF signaling network (top) and the GALECTIN signaling network (bottom). **(G)** The violin plot showed the expression of MIF signaling pathway-related genes (*MIF*, *ACKR3*, *CD74*, *CXCR4*, and *CD44*) in TC subtypes and across different cell types. **(H)** The heatmap showed the interactions among TC subtypes and different cell types in the GALECTIN signaling pathway.

To systematically analyze the cell-cell communication features mediated by different TC subtypes in the TME, we constructed a multicellular ligand-receptor interaction network based on CellChat analysis. First, **Figure 5A** displayed, from an overall perspective using circular plots, the communication network centered on C4 *TRIM31^+^* TCs as the source, quantifying the number and strength of interactions between this subtype and other cell types, which indicated its important role in the overall communication network. Considering the pivotal functions of macrophages in tumor immune regulation and inflammation, as well as their potential functional cooperation (87), we used macrophages as the source and visualized, via circular plots, the magnitude and frequency of macrophage interactions with each TC subtype. The results showed that communication between macrophages and C4 *TRIM31^+^* TCs was notably prominent, suggesting a potential close functional relationship between the two (**Figure 5B**). In **Figure 4**, the six hallmark-related pathways were suggested to be upstream of the MIF pathway; therefore, we subsequently examined the potential signaling network. Results indicated pronounced activity of the MIF signaling pathway in multiple cell populations. Specifically, in the outgoing signaling pattern, MIF and GALECTIN displayed high activity in C4 *TRIM31^+^* TCs, whereas in the incoming signaling pattern, MIF and GALECTIN were significantly enriched in macrophages, suggesting that intercellular communication might occur through the MIF and GALECTIN pathways (**Figure 5C**). To further characterize the transmission patterns of MIF signaling among cell populations, **Figures 5D–E** displayed, respectively, the overall network structure and directionality of the MIF-(CD74+CD44) pathway, the LGALS9-CD44 pathway, and the LGALS9-P4HB pathway using hierarchical and circular plots. In the hierarchical plots, when C4 *TRIM31^+^* TCs served as signal senders, interactions with macrophages as signal receivers were most prominent, suggesting that macrophages were likely the primary responders to MIF signaling. In the hierarchical plots of the LGALS9-CD44 and LGALS9-P4HB pathways, when macrophages served as signal senders, they communicated with multiple cell types via GALECTIN-mediated signaling; however, interactions with C4 *TRIM31^+^* TCs as signal receivers were most significant, indicating that C4 *TRIM31^+^* TCs were likely the main responders to GALECTIN signaling. Subsequent network role analysis revealed that in the MIF pathway, C4 *TRIM31^+^* TCs had the highest weight as senders, whereas macrophages had the highest weight as receivers and influencers. In the GALECTIN pathway, C4 *TRIM31^+^* TCs had the highest weight as senders and influencers, while macrophages had the highest weight as senders, mediators, and influencers, indicating that these cell types performed core signaling sending and receiving functions, respectively (**Figure 5F**). Notably, we performed further ligand-receptor pair analysis. **Figure 5G** displayed the expression distribution patterns of MIF, CD74, and CD44 among distinct cell types. The analysis revealed that these key molecules were predominantly expressed in TCs, particularly in C4 *TRIM31^+^* TCs, providing expression-level reinforcing the functional activity of the MIF signaling pathway within this TC subtype. The LGALS9-P4HB and LGALS9-CD44 pathways primarily occurred in the interactions from macrophages to C4 *TRIM31^+^* TCs. Macrophages consistently acted as key signal senders, and their interactions with C4 *TRIM31^+^* TCs via these pathways were the most prominent (**Figure 5H**).

### TF regulatory network construction and characterization in TC subtypes using SCENIC

**Figure 6:**
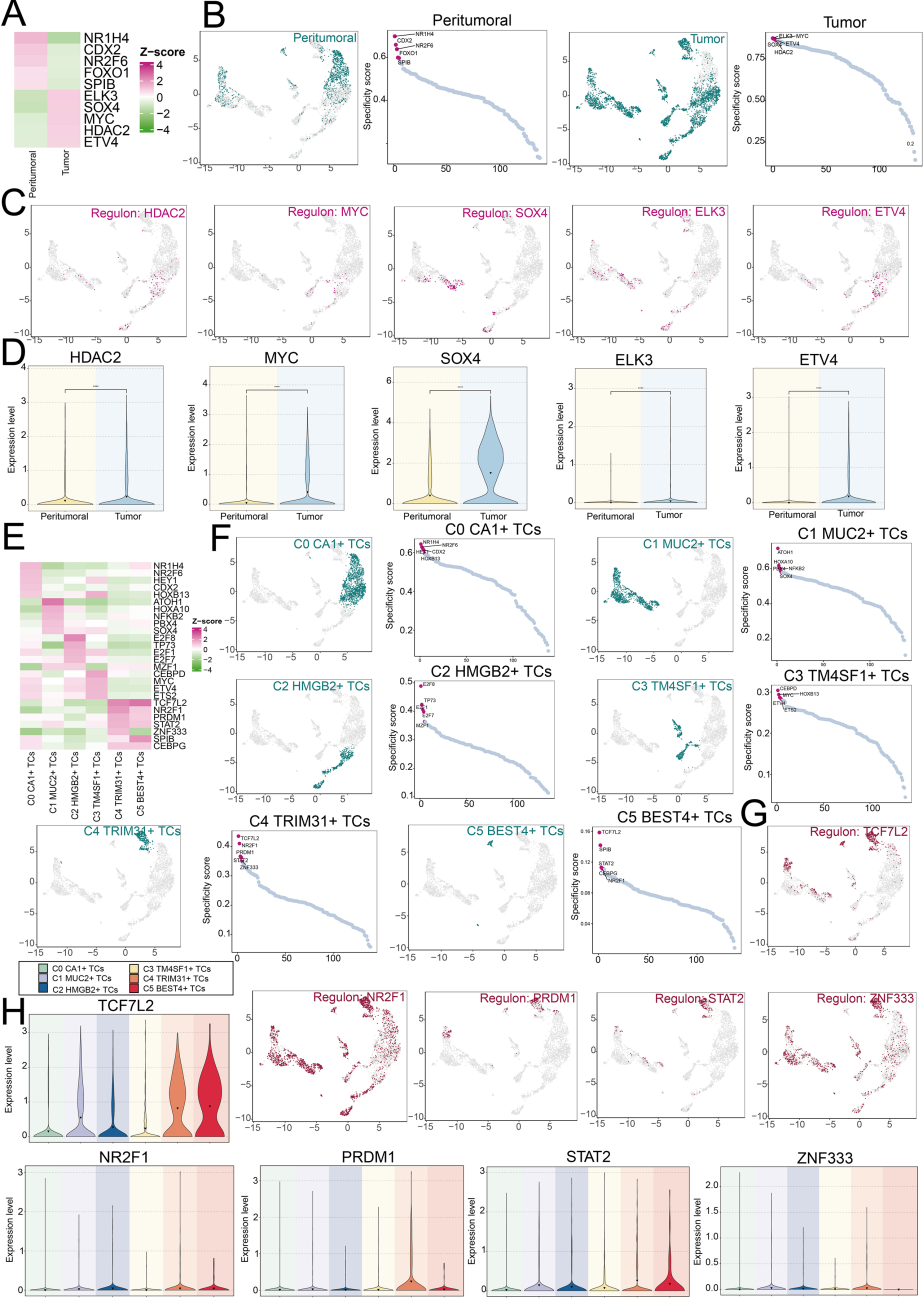
Distribution and heterogeneity of TF regulatory activity across tumors and their subtypes. **(A)** The heatmap showed the activity scores of the top 5 TFs in the peritumoral and tumor groups. **(B)** The scatter plots showed the activity scores of TFs in the peritumoral and tumor groups. **(C)** The UMAP plots showed the expression distribution of the regulatory activity of key TFs (HDAC2, MYC, SOX4, ELK3, and ETV4). **(D)** The violin plots showed the expression of key TFs in the peritumoral and tumor groups. **(E)** The heatmap showed the activity scores of the top 5 TFs in six TC subtypes. **(F)** The UMAP plots and scatter plots showed subtype-specific TFs in six TC subtypes. **(G)** The UMAP plots showed the expression distribution of the regulatory activity of key TFs in C4 *TRIM31^+^* TCs. **(H)** The violin plots showed the expression of key TFs across TC subtypes. ****P < 0.0001.

To systematically characterize the transcriptional regulatory functional and molecular features of various TC subtypes in CRC-related biological processes, we constructed a TF regulatory network of TCs based on SCENIC analysis and analyzed multiple aspects, including differential TF activity and subtype-specific regulatory patterns. At the TF level, **Figure 6A** displayed the top 5 active TFs in each group and compared their activity differences. These TFs exhibited distinct regulatory patterns between the peritumoral and tumor groups. Furthermore, **Figure 6B** projected the activity of these key TFs onto the UMAP plots, displaying their distribution patterns. We observed significant heterogeneity in TF activity across different groups. Meanwhile, the scatter plots quantitatively illustrated the dynamic changes in TF activity under different conditions, further validating their association with the group. **Figure 6C** further showed the distribution features of the top 5 TFs (HDAC2, MYC, SOX4, ELK3, and ETV4) in the tumor group, which exhibited clearly non-uniform expression patterns on the UMAP plots, indicating subtype-specific activation modes of the transcriptional regulatory network. At the quantitative level, **Figure 6D** illustrated the differences between the peritumoral and tumor groups; TF activities of HDAC2, MYC, SOX4, ELK3, and ETV4 expression was elevated in the tumor group compared with the peritumoral group, indicating enhanced activity of specific transcriptional regulatory programs. To further characterize the transcriptional regulatory features across TC subtypes, **Figure 6E** further illustrated a heatmap was generated to showcase the top 5 TFs exhibiting the highest levels of activity across each TC subtype. **Figure 6F** further showed the distribution and activity changes of TF regulons across different subtypes. Specifically, for C4 *TRIM31^+^* TCs, **Figures 6G, H** illustrated the distribution and expression of their top 5 TFs (TCF7L2, NR2F1, PRDM1, STAT2, and ZNF333), further supporting the transcriptional regulatory independence of this subtype. Different TFs exhibited clear subtype-preferential distributions among cell populations, further suggesting that TC subtypes might possess distinct functional differentiation at the level of transcriptional regulatory networks.

### Construction and validation of a prognostic model derived from C4 *TRIM31^+^* TCs

**Figure 7:**
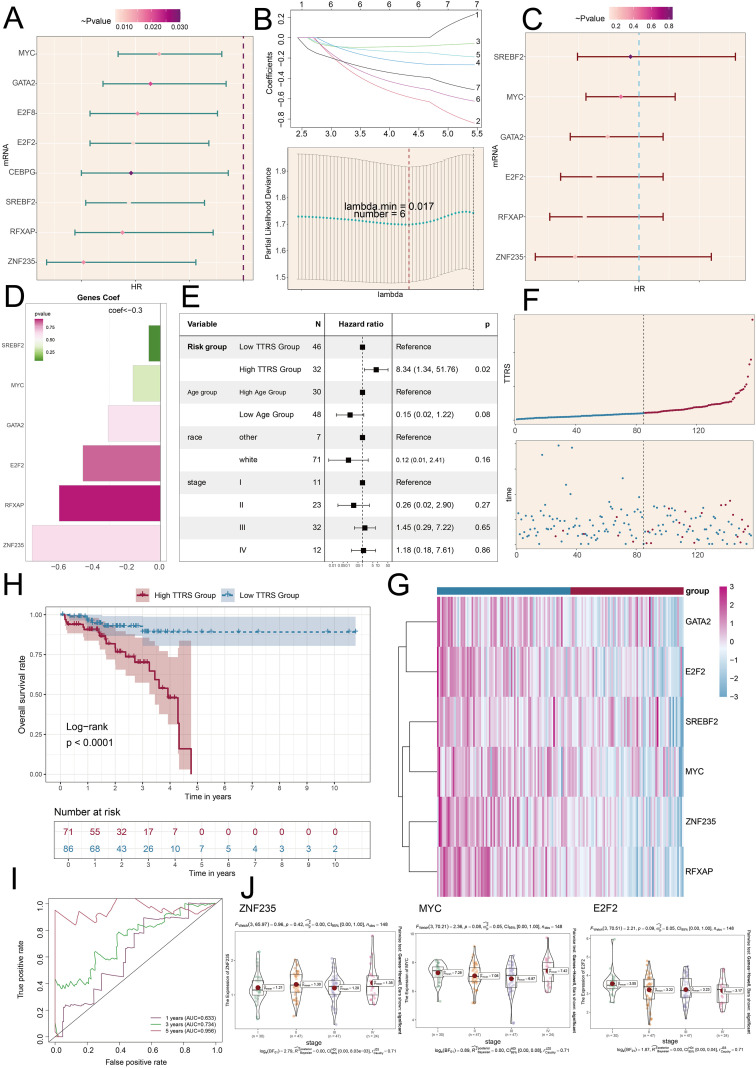
Construction and validation of the TTRS risk score model and its prognostic value and clinical characteristics. **(A)** The forest plot of univariate Cox regression analysis showed the hazard ratios and their 95% confidence intervals for eight genes significantly associated with prognosis (*MYC*, *GATA2*, *E2F8*, *E2F2*, *CEBPG*, *SREBF2*, *RFXAP*, and *ZNF235*), with the color gradient indicating the magnitude of *P values*. **(B)** The LASSO regression analysis was performed to select prognosis-related genes. The tenfold cross-validation for identifying the optimal parameter (λ) was shown (top), and the LASSO coefficient profiles were determined by the optimal λ (bottom). **(C)** The forest plot of multivariate Cox regression analysis showed the six prognostic genes (*SREBF2*, *MYC*, *GATA2*, *E2F2*, *RFXAP*, and *ZNF235*) that were ultimately included in the risk score model. **(D)** Coef values of the six genes selected by the LASSO Cox regression model were shown. **(E)** The forest plot of multivariate Cox analysis showed the clinicopathological factors, including risk group (high TTRS group vs low TTRS group), age group (high age group vs low age group), race (white vs other), and stage (I-IV). **(F)** The risk score curve for the high and low TTRS groups was shown (top), and the scatter plot showed the survival status (alive/deceased events) of the two groups over time (bottom). **(G)** The heatmap showed the differential expression of genes comprising the TTRS scoring model. **(H)** The Kaplan-Meier survival curve compared OS between patients in the high TTRS group (red) and the low TTRS group (blue). **(I)** The AUC values for predicting 1-year (AUC = 0.633), 3-year (AUC = 0.734), and 5-year (AUC = 0.956) survival outcomes in the TCGA cohort were shown. **(J)** The violin plots showed the expression of key risk genes (*ZNF235*, *MYC*, and *E2F2*) across different stages (I-IV).

Currently, reliable molecular biomarkers for CRC prognosis were urgently needed. To address this issue, Cox regression and Lasso Cox regression analyses were conducted on the top 100 TFs, leading to the development of a risk score model derived from C4 *TRIM31^+^* TCs (88). To begin with, univariate Cox regression was employed to perform an initial screening for prognostic candidates, resulting in eight genes, each exhibiting protective influence (**Figure 7A**). To control for multicollinearity, the candidate genes were further analyzed using Lasso-Cox regression, ultimately selecting 6 genes that, after λ validation, could be used to construct a scoring system (**Figure 7B**). Multivariate Cox regression subsequently identified six genes with independent prognostic significance. *ZNF235* and *RFXAP* were identified as the genes with the strongest effect on risk among the selected candidates (**Figure 7C**). Gene coefficient value<0 (**Figure 7D**), this was consistent with the above results, indicating that they were significant as protective factors related to prognosis. **Figure 7E** illustrated multivariate Cox regression for risk group, age, race, and stage, highlighting that the *TRIM31^+^* TCs risk group (TTRS) score independently predicted CRC patient outcomes (*P* < 0.05). **Figures 7F, G** depicted the TCGA cohort-wide distribution of TTRS scores. Patients were stratified into low- and high-TTRS groups based on the median score. Higher TTRS scores were associated with poorer prognosis and an increased number of deaths. Moreover, as the TTRS score increased, the transcriptional levels of GATA2, E2F2, SREBF2, MYC, ZNF235, and RFXAP progressively declined. Survival analysis using the Kaplan-Meier method revealed that patients with low TTRS scores exhibited significantly better overall survival compared with those in the high-TTRS group (**Figure 7H**). ROC survival curves in the TCGA cohort showed that the AUC values for 1-year, 3-year, and 5-year OS for CRC patients were 0.633, 0.734, and 0.956, respectively (**Figure 7I**). Furthermore, there were differences in gene expression levels among patients at different stages; for example, *ZNF235* expression levels were IV>II>I>III, *MYC* expression levels were IV>I>II>III, and *E2F2* expression levels were I>III>II>IV (**Figure 7J**).

### Immune infiltration profiling among different TTRS risk categories.

**Figure 8:**
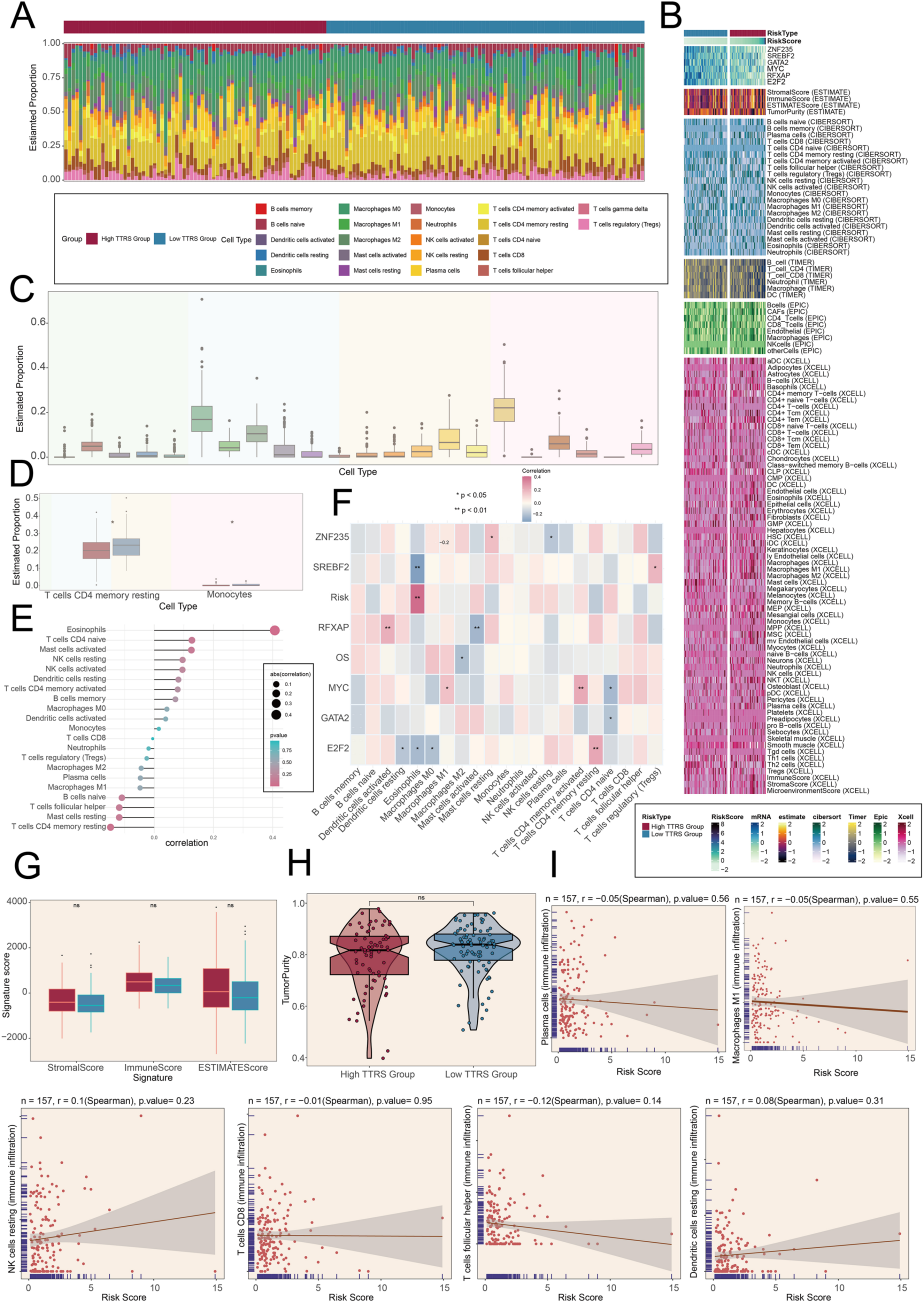
Immune cell infiltration and TME characteristics across TTRS groups. **(A)** The distribution of tumor-infiltrating immune cells between the high and low TTRS groups was analyzed using the CIBERSORT algorithm. **(B)** The differences between the high and low TTRS groups in model genes, stromal score, immune score, ESTIMATE score, tumor purity, and levels of immune cell infiltration were shown. **(C)** The box plot compared the proportions of 22 immune cell types between the high and low TTRS groups. **(D)** The box plot compared the proportions of T cells CD4 memory resting and monocytes between the high and low TTRS groups. **(E)** The lollipop plot showed the correlation between immune cells and TTRS group scores. **(F)** The heatmap showed the correlations among immune cells, genes comprising the TTRS scoring model, OS, and risk scores. **(G)** The differences in stromal score, immune score, and ESTIMATE score between high and low TTRS groups in CRC patients were analyzed using the ESTIMATE algorithm. **(H)** The violin plot showed the tumor purity levels between the high and low TTRS groups. **(I)** The correlation analysis between six key immune cell types and risk scores was performed.

Immune infiltration patterns in CRC samples were assessed via CIBERSORT and xCell, with stacked bar plot revealing variation in immune cell composition between patients with low and high TTRS scores (**Figure 8A**). To more comprehensively illustrate the immune microenvironment patterns under different TTRS scoring conditions, we employed heatmaps to illustrate differences in gene expression, matrix and immune scores, tumor purity, ESTIMATE scores, and immune cell infiltration levels across the two TTRS risk groups (**Figure 8B**). Furthermore, we assessed the estimated proportions of various immune cell populations in CRC, CD4 memory resting T cells were found to be the most prevalent, with M0 and M2 macrophages following closely behind in abundance (**Figure 8C**). Subsequently, we compared immune cell infiltration between the TTRS risk groups. The low-TTRS group showed a greater proportion of CD4 memory resting T cells and monocytes compared with the high-TTRS group, suggesting enhanced anti-tumor immune activity and reduced immunosuppressive effects in patients with lower TTRS scores (**Figure 8D**). We further assessed the correlations between immune cell populations and TTRS risk scores. Eosinophils, naïve CD4 T cells, and activated mast cells showed positive correlations with the TTRS score, whereas follicular helper T cells, resting mast cells, and CD4 memory resting T cells were negatively correlated, indicating distinct immune landscape patterns associated with higher versus lower risk scores (**Figure 8E**). Simultaneously, we examined the relationships between immune cell populations and the individual genes comprising the risk score model, OS, and the TTRS risk score (**Figure 8F**). The ESTIMATE algorithm was applied to calculate stromal scores, immune scores, and overall ESTIMATE scores for the low- and high-TTRS groups. The results indicated that the low-TTRS group exhibited lower stromal and immune scores compared with the high-TTRS group. In contrast, tumor purity tended to be higher in the low-TTRS group, although this difference did not reach statistical significance (**Figures 8G, H**). Finally, we depicted the immune correlations between the TTRS risk score and various immune cell subtypes, including M1 macrophages, CD8^+^ T cells, follicular helper T cells, plasma cells, resting dendritic cells, and resting NK cells (**Figure 8I**). Considering all factors, the findings suggested that patients with higher risk scores exhibit a shift toward an immunosuppressive microenvironment, characterized by reduced pro-inflammatory and adaptive immune cell activity alongside increased resting immune cell populations.

### TTRS risk stratification-related enrichment, gene mutations, and tumor mutation burden characteristics

**Figure 9:**
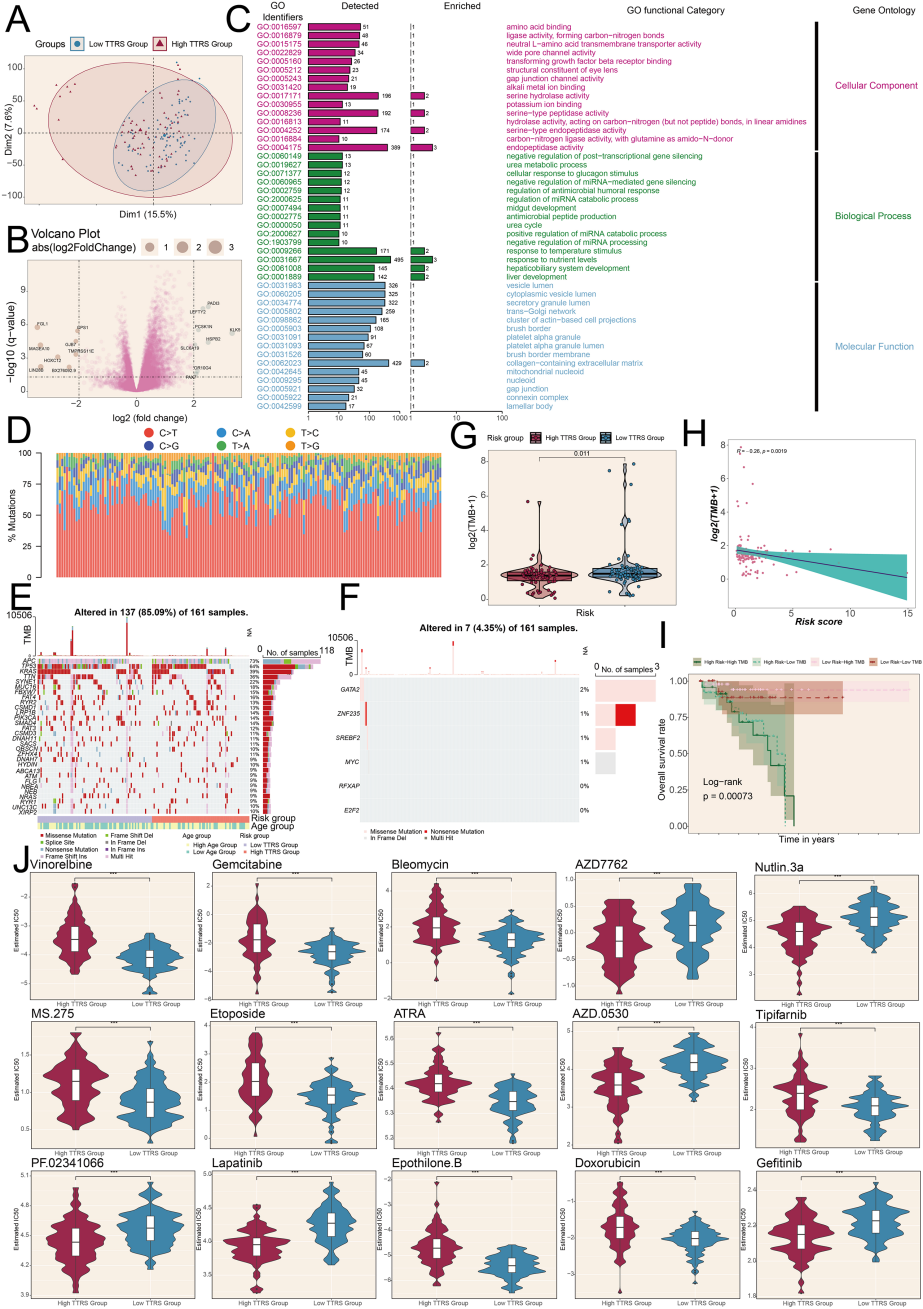
Functional enrichment, gene mutation, and drug sensitivity analyses across TTRS groups. **(A)** The PCA plot showed the distribution of the high and low TTRS groups along dim 1 and dim 2. **(B)** The volcano plot showed the DEGs between the high and low TTRS groups. **(C)** The GO functional enrichment analysis showed the DEGs between the high and low TTRS groups. **(D)** The stacked bar plot showed the mutation frequencies of six mutation types (C>A, C>G, C>T, T>A, T>C, and T>G) across all samples. **(E)** The waterfall plot showed the mutation status of the top 30 frequently mutated genes in 161 samples. The bar graph above showed the TMB of each sample, and the histogram on the right showed the mutation frequency percentage of each gene. **(F)** The waterfall plot showed the mutation profiles of six risk genes across samples. The bar graph above showed the mutation burden of each gene, and the histogram on the right showed the mutation types of each gene. **(G)** The violin plot showed the differences in TMB between the high and low TTRS groups. **(H)** The correlation analysis between risk scores and TMB was performed. **(I)** The Kaplan-Meier survival curve showed the survival outcomes of four groups: low TTRS high TMB, low TTRS low TMB, high TTRS high TMB, and high TTRS low TMB. **(J)** The violin plots showed the differences in drug sensitivity between the high and low TTRS groups. *** < 0.001.

To evaluate broad distinctions in prognostic gene expression across CRC samples, we projected the data into principal component space. PCA plot demonstrated a clear separation between the low- and high-TTRS groups, reflecting distinct transcriptional profiles associated with different risk scores (89) (**Figure 9A**). To explore functional differences between the TTRS groups, we conducted gene enrichment analysis. Volcano plot results highlighted pronounced differential expression, with *KLK5*, *PADI3*, and *HSPB2* significantly upregulated, whereas *FGL1*, *MAGEA10*, and *LIN28B* were markedly downregulated in one group compared with the other (**Figure 9B**). To further explore the biological implications of these results, we performed GO enrichment analysis targeting molecular functions, biological processes, and cellular components, highlighting the involvement of DEGs in key regulatory pathways (**Figure 9C**). To understand the mutated genes within CRC, we analyzed the mutations present in cells of the low TTRS and high TTRS groups, revealing that C>T mutations accounted for the highest proportion (**Figure 9D**). **Figure 9E** showed the mutation status of the top 30 most frequently mutated genes in 161 samples, with missense mutation being the predominant mutation type, and *APC* displaying the highest mutation frequency. Furthermore, **Figure 9F** illustrated mutation spectrum of the six genes used in the risk model, revealing mutations in only 7 of the 161 cases, with *GATA2*, *ZNF235*, and *SREBF2* exhibiting the highest mutation rates, where missense mutations constituted the most frequent form of genomic alteration. Following this, we compared the tumor mutational burden between the low-TTRS and high-TTRS groups. Remarkably, the TMB was elevated in the low-TTRS group relative to the high-TTRS group, with the difference reaching statistical significance (*P* < 0.05) (**Figure 9G**). In addition, a significant inverse relationship was observed between TMB and risk score (**Figure 9H**). To investigate the association between TMB, TTRS group, and OS, we combined TMB and TTRS group scores to divide patients into four distinct groups: high-TTRS high-TMB, high-TTRS low-TMB, low-TTRS high-TMB, and low-TTRS low-TMB. As illustrated in **Figure 9I**, patients with low TTRS and high TMB exhibited the highest overall survival, followed by those with low TTRS and low TMB. In contrast, no significant differences in OS were observed between the high-TTRS/high-TMB and high-TTRS/low-TMB groups, providing further support for our earlier observations.

### Analysis of drug sensitivity

Finally, to evaluate the differential drug sensitivity between the low- and high-TTRS groups, we employed the “RRophetic” R package to predict patient-specific responses using pharmacogenomic data from the GDSC database. The analysis revealed notable differences in drug sensitivity between the low- and high-TTRS groups. Gemcitabine, Etoposide, ATRA, Bleomycin, and Tipifarnib exhibited higher predicted half-maximal inhibitory concentrations (IC50) in the high-TTRS group, whereas Gefitinib, Nutlin-3a, and AZD7762 showed elevated IC50 values in the low-TTRS group. Collectively, these findings suggest that differences in the immune microenvironment and tumor characteristics are closely associated with therapeutic response (**Figure 9J**).

### *TRIM31* knockdown inhibited CRC cell proliferation and migration while inducing apoptosis

**Figure 10:**
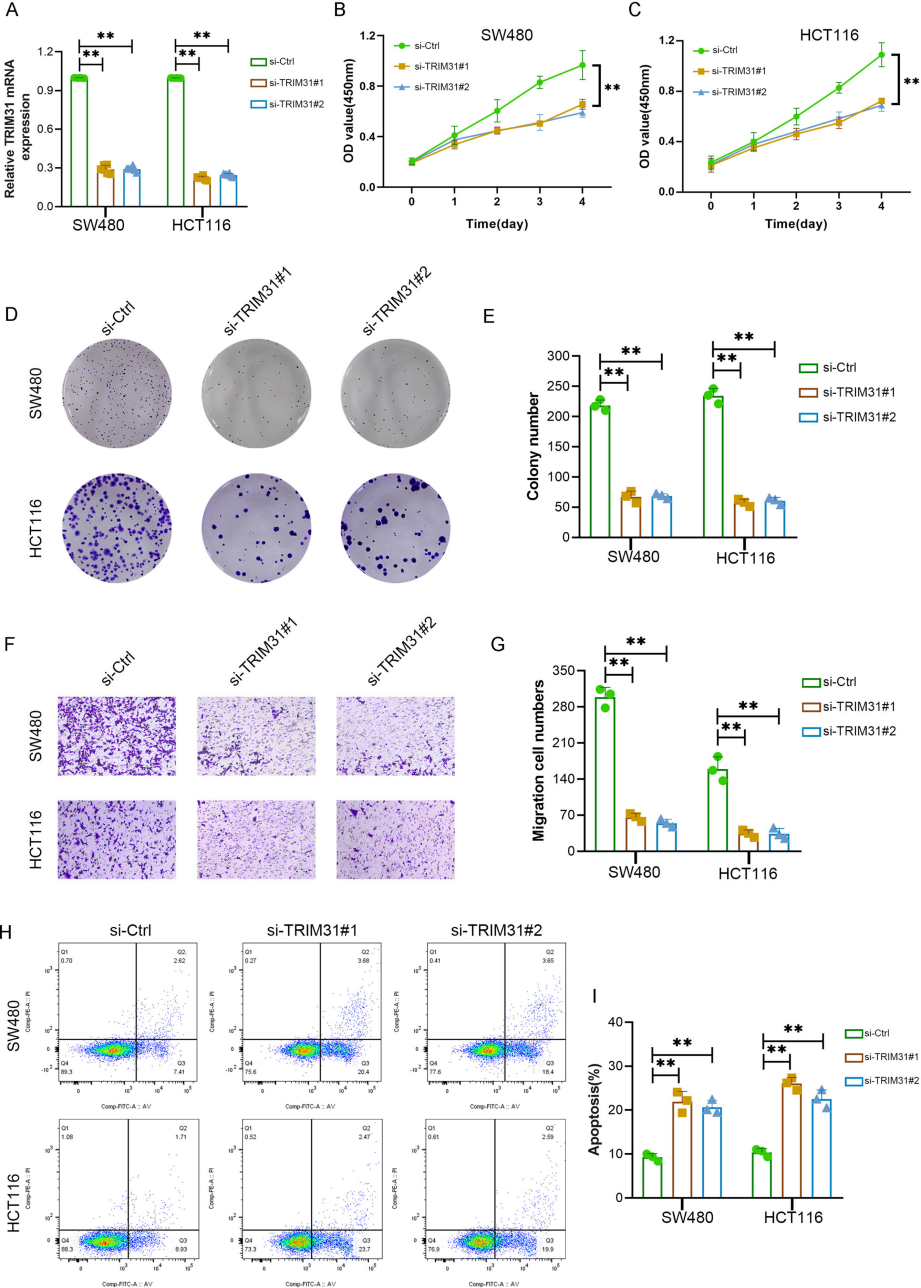
Effects of *TRIM31* knockdown on proliferation, migration, and apoptosis in CRC cells. **(A)** qRT-PCR analysis showing relative *TRIM31* mRNA expression levels in SW480 and HCT116 cells after transfection with si-Ctrl, si-*TRIM31*#1, or si-*TRIM31*#2. **(B, C)** CCK-8 assays assessing cell proliferation at indicated time points in SW480 **(B)** and HCT116 **(C)** cells following *TRIM31* knockdown. **(D)** Representative images of colony formation assays in SW480 and HCT116 cells after *TRIM31* silencing. **(E)** Quantification of colony numbers demonstrating reduced clonogenic capacity in si-*TRIM31* groups. **(F)** Representative images of transwell migration assays in SW480 and HCT116 cells. **(G)** Quantification of migrated cell numbers showing significantly decreased migration after *TRIM31* knockdown. **(H)** Representative Annexin V/PI flow cytometry plots showing apoptosis distribution in SW480 and HCT116 cells. **(I)** Statistical analysis of apoptotic rates indicating increased apoptosis following *TRIM31* silencing. Data were presented as mean ± SD from three independent experiments. **P < 0.01 versus si-Ctrl.

To explore the role of *TRIM31* in CRC progression, SW480 and HCT116 cells were transfected with two independent siRNAs targeting *TRIM31* (si-*TRIM31*#1 and si-*TRIM31*#2). qRT-PCR results demonstrated that transfection with si-*TRIM31*#1 or si-*TRIM31*#2 effectively suppressed *TRIM31* expression in SW480 and HCT116 cells compared to controls (**Figure 10A**), validating the efficiency of *TRIM31* silencing. Next, we assessed the impact of *TRIM31* knockdown on CRC cell proliferation using CCK-8 assays. *TRIM31* knockdown significantly suppressed the time-dependent increase in OD450 values in both SW480 and HCT116 cells relative to control cells (**Figures 10B, C**), indicating impaired proliferative capacity. To further evaluate long-term clonogenic potential, colony formation assays were performed. Representative images showed that *TRIM31* silencing substantially reduced colony formation ability in both CRC cell lines (**Figure 10D**). Colony formation analysis showed markedly fewer colonies following *TRIM31* silencing relative to controls (**Figure 10E**). Considering that TC migration plays a critical role in cancer progression and metastasis, transwell migration assays were conducted to assess the effect of *TRIM31* knockdown on cell motility. As shown in **Figures 10F, G**, depletion of *TRIM31* caused a notable reduction in the number of migrated cells in both SW480 and HCT116 cells, suggesting that *TRIM31* was positively associated with the migratory capacity of CRC cells. Finally, Annexin V/PI flow cytometry analysis demonstrated that *TRIM31* knockdown markedly increased apoptotic cell populations compared with the control group in both cell lines (**Figures 10H, I**), indicating that *TRIM31* contributes to CRC cell survival. Collectively, these data demonstrate that *TRIM31* facilitates cell growth and migratory capacity while restraining apoptotic processes, underscoring its pro-tumorigenic role in CRC.

## Discussion

CRC is a major contributor to the global burden of gastrointestinal malignancies, ranking as the third most common cancer worldwide and causing substantial morbidity and mortality (90, 91). Despite the wide range of treatment options for CRC, encompassing surgical resection, chemotherapy, radiation therapy, targeted treatment, and immunotherapeutic approaches, the practical implementation of these methods is still hindered by multiple limitations (92–94). Significant intratumoral and interpatient heterogeneity contributes to variable treatment outcomes, reducing the overall effectiveness of standard therapies. In addition, the emergence of drug resistance following initial treatment poses a critical barrier, often leading to tumor progression (95). In the same vein, the intricate TME further complicates treatment, with immunosuppressive populations such as TAMs and regulatory T cells promoting immune escape and inhibiting TC function via immunosuppressive factors and metabolic products (96). Besides, CRC cells can exploit metabolic reprogramming, including increased glycolysis, glutamine metabolism, and fatty acid biosynthesis, to adapt to microenvironmental changes, maintain their growth advantage, and potentially develop drug resistance (97, 98). Therefore, identifying novel strategies for the prevention and treatment of CRC is urgently needed.

Through the integration of single-cell transcriptomic profiling, regulatory network reconstruction, and cell-cell communication analysis, this study characterized a multi-layered cancer-immunity regulome in CRC. Here, we characterized the cell types that played key roles in CRC progression. The heterogeneity of TCs was particularly noteworthy, we characterized six CRC TC subtypes, namely C0 *CA1^+^ TCs*, C1 *MUC2^+^ TCs*, C2 *HMGB2^+^ TCs*, C3 *TM4SF1^+^ TCs*, C4 *TRIM31^+^ TCs*, and C5 *BEST4^+^* TCs, highlighting the intricate heterogeneity of CRC. Within these TCs, C4 *TRIM31^+^* TCs exhibited immune-related gene expression and functions, and this subtype was more prevalent in the tumor group of CRC, reflecting its expected involvement in tumor advancement. We additionally noted that C4 *TRIM31^+^* TCs were more inclined to reside in the G1 phase, suggesting a preferential cell cycle distribution toward a preparatory or relatively quiescent state rather than active proliferation. This phase represented a critical restriction point at which cells determined whether to proceed into the cell cycle or enter the G0 quiescent state under unfavorable conditions (99, 100). Notably, *TRIM31* was identified as a key regulatory node that interconnected transcriptional activity, metabolic remodeling, and tumor-immune crosstalk, underscoring its pivotal role in coordinating the regulatory dynamics of the TME.

The study also revealed significant differences among TC subtypes in biological processes and metabolic profiles. Integrative analyses indicated that C4 *TRIM31^+^* TCs were predominantly enriched in biological processes associated with mitochondrial oxidative phosphorylation-driven metabolic reprogramming, ROS-related damage repair and oncogenic processes, host-exogenous factor interactions, and remodeling of cell-cell junction structures. Therefore, we inferred that C4 *TRIM31^+^* TCs might have promoted tumor progression through a combination of metabolic adaptation, oxidative stress regulation, and cellular structural remodeling. Notably, C4 *TRIM31^+^* TCs might have participated in tumor immune regulation by mimicking antigen-presenting behaviors of immune cells. TCs were typically spatially divided into tumor core and tumor margin regions, with cells located at the tumor margin being more likely to directly contact and interact with immune cells (101). Within this microenvironment, some TCs might have exhibited an “immune mimicry” phenomenon by expressing immune cell-like molecular features or antigen-presentation-related molecules, making them difficult to be effectively recognized by immune cells during immune surveillance. This strategy resembled a “camouflage” mechanism, enabling TCs to act like “spies” within the immune system, thereby reducing immune clearance efficiency and ultimately promoting immune escape and the establishment of an immunosuppressive microenvironment. In comparison, the remaining TC subtypes exhibited functionally diverse characteristics.

We then explored the cell differentiation paths and stem cell-like properties of CRC TC subtypes, enabling a more comprehensive view of cellular dynamics and functional heterogeneity across the TME. CytoTRACE and Monocle analyses suggested a potential developmental trajectory. Compared with other TC subtypes, C4 *TRIM31^+^* TCs were predominantly located at the late stage of the pseudotime trajectory, indicating that they may represent a relatively later or more mature TC state. TCs during CRC initiation and progression did not exist as discrete states but exhibited continuous transcriptional changes, reflecting dynamic transitions during their evolution. Our Slingshot results indicated that both lineages 1 and 2 originated from C2 *HMGB2^+^* TCs, suggesting that TCs characterized by high *HMGB2* expression might have possessed certain progenitor-like features, enabling differentiation into TC subtypes with distinct functional states. Lineage 2 underwent a series of differentiation events, ultimately giving rise to C4 *TRIM31^+^* TCs. Dynamic gene expression analysis revealed a progressive upregulation of *TRIM31* along the pseudotime trajectory, consistent with its enrichment in the late pseudo-temporal stage. This time-dependent expression pattern suggested that *TRIM31* may have participated in the transcriptional reprogramming of TCs during their developmental trajectory.

Prior research had indicated that *TRIM31* participated in the regulation of inflammatory responses and multiple tumor-related signaling pathways. In single-cell analyses (102, 103), C4 *TRIM31^+^* TCs typically exhibited active cytokine signaling, strong communication with immune cells, and involvement in tumor-immune interactions. Therefore, they could be defined as an immune-interacting tumor epithelial subtype. This suggested that C4 *TRIM31^+^* TCs might have possessed specific biological functions within the TME and indicated that this subtype could have acted as a critical driver of cancer progression and intratumoral heterogeneity. To further assess and substantiate the contribution of C4 *TRIM31^+^* TCs to CRC, we retrieved and scored the top 50 hallmark pathways and further analyzed the functional heterogeneity of TC subtypes. Moreover, C4 *TRIM31^+^* TCs were found to be significantly enriched in a range of immune- and inflammation-associated pathways, including TNFA signaling via NFκB, IL6 JAK STAT3 signaling, and inflammatory response-related pathways. These pathways had been recognized in the TME as key axes driving chronic inflammation and immune regulation. Previous studies demonstrated that TNFA signaling via NFκB and IL6 JAK STAT3 signaling could promote sustained activation of inflammatory factors in CRC and participated in the establishment of a tumor-associated immune microenvironment by regulating the expression of various cytokines and chemokines (104, 105). In addition, the activation of the complement system and inflammatory response pathways was considered to be closely associated with immune cell recruitment and the amplification of inflammatory reactions (106). Additionally, apart from the pathways noted above, C4 *TRIM31^+^* TCs showed significant enrichment in tumorigenesis-related signaling pathways, including hypoxia, KRAS signaling up, and PI3K AKT MTOR signaling. Hypoxia, as a hallmark of solid tumors, could promote TC survival and the formation of an immunosuppressive microenvironment through HIF-1α-mediated transcriptional regulation (107–109). Meanwhile, the KRAS and PI3K-AKT-mTOR signaling pathways, as classical oncogenic axes in CRC, drove TC proliferation and metabolic reprogramming, while simultaneously enhancing the expression of inflammatory factors and immune-regulatory molecules (110–112). It was noteworthy that cytokine genes, immune ligand genes, and macrophage-related signaling pathways were under the regulation of *TRIM31*, and certain pathways functioned as upstream regulators of the MIF signaling pathway, thus affecting MIF expression or secretion. Meanwhile, immune-related pathways, including inflammatory responses and interferon signaling, were additionally shown to facilitate MIF secretion, thereby establishing a positive inflammatory feedback loop and regulating immune cell functions (113, 114).

MIF was a critical inflammatory cytokine involved in tumorigenesis, immune regulation, and inflammatory responses. In CRC, MIF was secreted by TCs and activated multiple downstream inflammatory and survival signaling pathways by binding to receptors on immune cells, such as CD74 and its co-receptor CD44. This process modulated immune cell functions and promoted the formation of the TME, particularly by influencing macrophage polarization and the establishment of an immunosuppressive microenvironment (115, 116). Based on these results, we further focused on the MIF signaling pathway in cell–cell communication, and an analysis was carried out to assess the potential interactions between C4 *TRIM31^+^* TCs and other cell types, including macrophages. In tumors, MIF signaling was found to modulate macrophage polarization. M1 macrophages exhibited anti-tumor and pro-inflammatory functions, whereas M2 macrophages displayed immunosuppressive and pro-tumor activities. MIF promoted the polarization of TAMs toward an M2-like phenotype, leading to increased secretion of immunosuppressive factors such as IL-10 and TGF-β, thereby facilitating the establishment of an immunosuppressive TME. However, the effects of MIF were microenvironment-dependent: it promoted tumor progression during chronic tumor inflammation, enhanced immune responses during early immune activation, and participated in immune surveillance during macrophage recruitment (117). *TRIM31* was suggested to primarily maintain communication and immune surveillance between TCs and macrophages, rather than directly promoting the polarization of immunosuppressive macrophages. C4 *TRIM31^+^* TCs were predicted to participate in MIF-related crosstalk based on CellChat, reducing M2-like TAMs, while it potentially suppressed phagocytic activity, decreased the clearance of TCs, and facilitated tumor immune evasion by regulating the secretion of inflammatory and immune factors. Further ligand-receptor analysis indicated that MIF-(CD74+CD44) interactions mediated intercellular communication between C4 *TRIM31^+^* TCs and macrophages, subsequently influencing macrophage functional changes. This signaling axis was considered in multiple tumors to regulate macrophage recruitment and polarization and facilitate the development of an immunosuppressive TME (118, 119), supporting the existence of a critical signaling axis between C4 *TRIM31^+^* TCs and macrophages.

Apart from MIF signaling, moreover, GALECTIN-mediated signaling played an important role in macrophage-TC interactions. Unlike MIF signaling, which was mainly transmitted from TCs to macrophages, GALECTIN signaling exhibited an opposite directionality: macrophages primarily acted as signal senders, whereas C4 *TRIM31^+^* TCs served as the main signal receivers. Specifically, ligand-receptor pairs such as LGALS9-P4HB and LGALS9-CD44 showed significant interactions between macrophages and C4 *TRIM31^+^* TCs, potentially enabling macrophages to evade recognition and phagocytosis of TCs. Proteins of the GALECTIN family had been demonstrated to play essential roles in regulating tumor immune evasion, T cell function suppression, as well as TC adhesion and migration (120, 121). Thus, macrophages transmitted regulatory signals to TCs via GALECTIN signaling, which potentially further influenced the biological behavior of TCs.

TFs played a central role in regulating gene expression and cellular behaviors. Therefore, for C4 *TRIM31^+^* TCs, we used SCENIC analysis to reveal significant TF regulatory heterogeneity between groups and subtypes at the transcriptional regulatory level. ELK3, SOX4, MYC, HDAC2, and ETV4 exhibited higher activity in the tumor group, and most of these TFs were closely involved in tumor development, cellular proliferation, and epithelial-mesenchymal transition (122–127). For example, MYC was considered a classical oncogenic TF that drove metabolic reprogramming and proliferation in TCs; SOX4 and ETV4 were implicated in regulating cell motility, invasion, and tumor advancement in various cancers; HDAC2, as an epigenetic modulator, influenced the expression levels of multiple tumor-related genes by regulating chromatin states (123, 124, 127–129). Therefore, these TFs likely represented core transcriptional programs commonly activated in CRC TCs. Further analysis revealed that C4 *TRIM31^+^* TCs exhibited upregulation of multiple TFs, notably the top 5: TCF7L2, NR2F1, PRDM1, STAT2, and ZNF333. TCF7L2 served as a critical transcriptional regulator within the Wnt/β-catenin signaling pathway, involved in regulating intestinal epithelial homeostasis and contributing to CRC progression (130, 131); STAT2 participated in interferon signaling and immune response regulation (132); PRDM1 was associated with stemness regulation and tumor growth (133, 134); NR2F1 was related to tumor dormancy (135); and ZNF333 was involved in RNA transcription (136). The enrichment of these TFs suggested that C4 *TRIM31^+^* TCs not only possessed a distinct TC state but also potentially contributed to tumor immune regulation and interactions within the TME.

A predictive risk model based on C4 *TRIM31^+^* TCs was established and validated by combining multi-omics data with clinical prognostic parameters. The results indicated that *SREBF2*, *MYC*, *GATA2*, *E2F2*, *RFXAP*, and *ZNF235* exhibited potential protective effects. Based on the optimal cutoff value of the TTRS risk score, patients were classified into high- and low-TTRS groups, with the high-TTRS group exhibiting significantly poorer overall survival. Further analyses were performed to assess how C4 *TRIM31^+^* TCs shape the tumor immune microenvironment. Distinct patterns in immune cell distribution suggested that the high TTRS group might have evaded immune surveillance through immune escape mechanisms, thereby promoting tumor progression. Further analysis revealed that macrophages M1, CD8^+^ T cells, follicular helper T cells, and plasma cells were negatively correlated with the risk score; these cell types were typically associated with immune activation (137–140). Resting dendritic cells and resting NK cells were positively correlated with the risk score; these cell types were typically associated with immune suppression (141, 142). The results suggested that the high-TTRS group was characterized by a more immunosuppressive TME, which might facilitate immune escape of TCs.

Enrichment analysis revealed that, in terms of cellular component annotations, marked enrichment was observed in serine-type peptidase activity, serine-type endopeptidase activity, and endopeptidase activity. Extracellular matrix remodeling was observed to affect immune responses within the TME (143). At the Biological Process level, response to temperature stimulus, response to nutrient levels, hepaticobiliary system development, and liver development were significantly enriched. These stress response and metabolic adaptation processes suggested that the TCs had acquired adaptive capacity under hypoxia, nutrient deprivation, or environmental stress, representing a “survival advantage” (144). The reactivation of developmental programs indicated that the cells had obtained a development-like state. At the Molecular Function level, collagen-containing extracellular matrix was significantly enriched, indicating enhanced proteolysis and enzymatic activity, as well as increased tumor invasiveness and remodeling capability within the TME (145). Furthermore, differences in TMB represented another important factor affecting patient prognosis, as these mutation sites could lead to distinct tumor outcomes. Several studies demonstrated that elevated TMB was positively correlated with improved survival in CRC patients (146). These observations were suggestive of consistent with our observations. Finally, a comprehensive drug sensitivity analysis was conducted, implying that individuals in the high-risk TTRS group exhibited greater responsiveness to agents such as Vinorelbine, Gemcitabine, and Bleomycin. These findings not only highlight potential therapeutic targets but also provide insights into underlying mechanisms of drug resistance, offering a basis for future drug repurposing and combination treatment strategies.

The conclusions described above were further supported by evidence obtained from *in vitro* experiments. Silencing of *TRIM31* markedly suppressed CRC cell proliferation and colony-forming capacity. Furthermore, the significant decrease in colony-forming ability and the suppression of cell migration after *TRIM31* knockdown further support its role in maintaining long-term tumorigenic potential. Importantly, *TRIM31* knockdown significantly increased apoptosis in CRC cells. This anti-apoptotic effect might be achieved by regulating pathways related to inflammation and immune responses, notably TNFA/NFκB and IL6-JAK-STAT3 signaling pathways, which were known to enhance cell survival and anti-apoptotic capacity. These findings collectively highlighted *TRIM31* as an essential modulator of the malignant phenotype of CRC. These experimental results functionally substantiated the regulatory network predicted by computational analyses, effectively bridging the gap between in silico inference and biological mechanism.

This study offered novel targets and potential strategies for precision therapy in CRC, especially indicating that combining metabolic pathway-targeted therapy with immunomodulatory treatment offered significant advantages. Taken together, our findings revealed an integrated regulatory framework whereby genomic variations and transcriptional reprogramming orchestrated TC state transitions, which in turn modulated tumor-immune interactions via ligand-receptor signaling. This hierarchical, multi-layered cascade was ultimately associated with immune dysfunction and facilitated tumor immune escape, constituting a critical component of the cancer-immunity regulome in CRC. However, the study had several limitations. First, the relatively limited sample size and restricted geographic representation may not have fully captured the extensive heterogeneity of CRC, particularly across different molecular subtypes. Second, while experimental results *in vitro* suggested a regulatory effect of *TRIM31*, additional *in vivo* validation was required to confirm its function within the intact TME. Moreover, the complexity of sample acquisition, along with time-consuming procedures for collection, processing, and staining, prevented the completion of all experimental analyses within a short timeframe. Therefore, future studies should further refine macrophage classification and determine which specific subpopulations are modulated by C4 *TRIM31^+^* TCs. Finally, although a prognostic risk model was established, its clinical applicability still required validation in larger, independent cohorts and prospective clinical studies to ensure robustness and translational value. This was consistent with the broader CRC biomarker literature, in which many promising candidate markers remained limited by small study populations, potential selection bias, and insufficient multicenter prospective validation before clinical implementation (147).

## Conclusion

Using scRNA-seq, this study comprehensively characterized the pronounced heterogeneity of CRC and identified C4 *TRIM31^+^* TCs as a distinct immune-related tumor epithelial subtype. Using an integrative analytical strategy, we characterized a multi-level cancer-immunity regulome that integrates transcriptional control, tumor evolutionary dynamics, and cell-cell communication. Importantly, *TRIM31* was defined as a key regulatory node that promoted TC proliferation and migration, inhibited apoptosis, and coordinated the interaction between TC and the immune microenvironment. *In vitro* experimental validation revealed that *TRIM31* acts as a key mediator influencing the proliferation, migration, and apoptosis of CRC TCs. Moreover, the risk score TTRS model derived from these findings showed robust clinical predictive capability. Overall, this research offered novel insights into the regulatory mechanisms underlying tumor-immune interactions in CRC and identifies potential targets for precision immunotherapy. Future studies should further investigate the molecular characteristics and clinical translational potential of *TRIM31* across different CRC subtypes and further elucidate the mechanisms and prognostic therapeutic strategies critical to CRC progression.

## Data Availability

The original contributions presented in the study are included in the article/**Supplementary Material**. Further inquiries can be directed to the corresponding author/s.

## Supplementary material

Supplementary Figure 1 Unsupervised clustering and CNV analysis of cells from CRC and adjacent normal tissues. **(A)** The UMAP plot showed the 28 cell clusters identified by unsupervised single-cell clustering, with each cluster color-coded. **(B)** The inferCNV analysis heatmaps compared CNV profiles between fibroblasts and epithelial cells, with red representing amplification and blue representing deletion. **(C)** The violin plot showed the CNV scores between fibroblasts and epithelial cells.
