# Supplementary figures and images for: Integrative single-cell analysis reveals TRIM31+ colorectal tumor cells orchestrating macrophage crosstalk within the cancer-immunity regulome
Source: Front Immunol. 2026 May 29;17:1848654. doi: 10.3389/fimmu.2026.1848654 (PMC13260437; doi:10.3389/fimmu.2026.1848654)

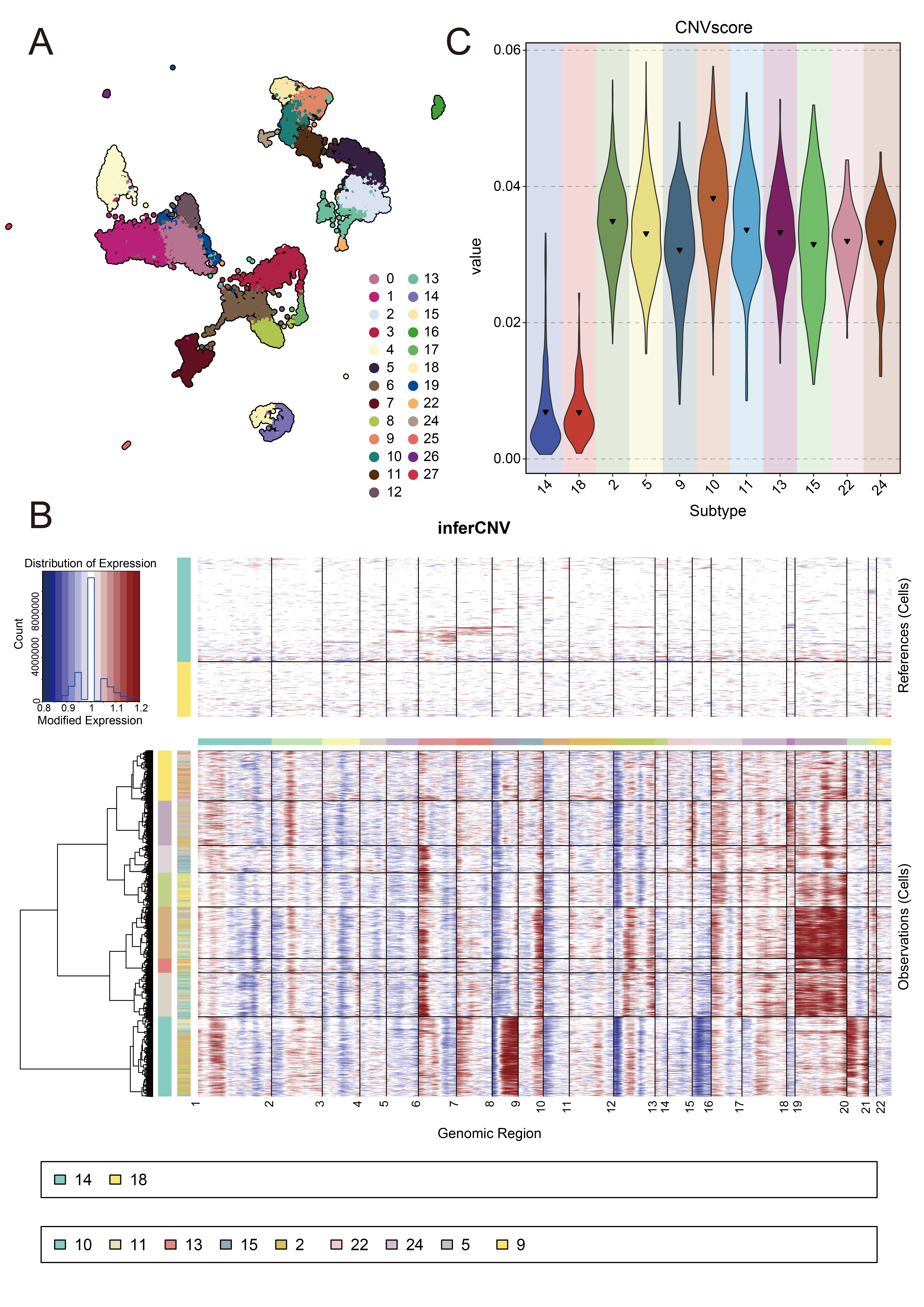

Supplement: Supplementary Figure 1 — Unsupervised clustering and CNV analysis of cells from CRC and adjacent normal tissues. (A) The UMAP plot showed the 28 cell clusters identified by unsupervised single-cell clustering, with each cluster color-coded. (B) The inferCNV analysis heatmaps compared CNV profiles between fibroblasts and epithelial cells, with red representing amplification and blue representing deletion. (C) The violin plot showed the CNV scores between fibroblasts and epithelial cells. [file Image1.jpeg]
