# Supplementary material for: Integrative single-cell analysis reveals TRIM31+ colorectal tumor cells orchestrating macrophage crosstalk within the cancer-immunity regulome
Source: Front Immunol. 2026 May 29;17:1848654. doi: 10.3389/fimmu.2026.1848654 (PMC13260437; doi:10.3389/fimmu.2026.1848654)
Supplement: Supplementary file 2 [file Table1.docx]

**Supplementary Table S1**

| **Gene** | **Type** | **Name** | **Sequence (5′→3′)** |
| --- | --- | --- | --- |
| TRIM31 | siRNA | si-TRIM31#1 | GCUCAGAGAUCUUCGAGAUTT |
| TRIM31 | siRNA | si-TRIM31#2 | CCAGAAAGCUACUGGAAUATT |
| TRIM31 | qPCR | Forward | AGCAGCAGTTTGTGGAGATG |
| TRIM31 | qPCR | Reverse | TGGTAGGTGAGGTTGGTGAT |
| GAPDH | qPCR | Forward | GGAGCGAGATCCCTCCAAAAT |
| GAPDH | qPCR | Reverse | GGCTGTTGTCATACTTCTCATGG |
